# Pediatric Emergency Medicine Simulation Curriculum: Vitamin K Deficiency in the Newborn

**DOI:** 10.15766/mep_2374-8265.11078

**Published:** 2021-01-25

**Authors:** Elizabeth Sanseau, Leah H. Carr, Jennifer Case, Khoon-Yen Tay, Anne Ades, Kesi Yang, Hannah Huang, Anna Bustin, Grace Good, Shannon Gaines, Julie Augenstein, Daisy Ciener, Jean Pearce, Jennifer Reid, Kimberly Stone, Rebekah Burns, Anita Thomas

**Affiliations:** 1 Fellow, Pediatric Emergency Medicine, Children's Hospital of Philadelphia; 2 Fellow, Department of General Pediatrics, Division of Neonatology, Children's Hospital of Philadelphia; 3 Resident, Department of General Pediatrics, University of Washington School of Medicine and Seattle Children's Hospital; 4 Associate Professor, Clinical Pediatrics, Division of Emergency Medicine, Children's Hospital of Philadelphia; 5 Professor, Clinical Pediatrics, Division of Neonatology, Children's Hospital of Philadelphia; 6 Assistant Professor, Clinical Pediatrics, Division of Neonatology, Children's Hospital of Philadelphia; 7 Pharmacist, Department of General Pediatrics, Division of Emergency Medicine, Children's Hospital of Philadelphia; 8 Residency Preceptor, Pharmacy Residency Program, and Clinical Pharmacy Specialist, Neonatal/Infant Intensive Care, Children's Hospital of Philadelphia; 9 Simulation Specialist, Division of Emergency Medicine, Children's Hospital of Philadelphia; 10 Education Nurse Specialist, Division of Emergency Medicine, Children's Hospital of Philadelphia; 11 Assistant Professor, Clinical Pediatrics, Division of Emergency Medicine, Phoenix Children's Hospital; 12 Assistant Professor, Clinical Pediatrics, Division of Pediatric Emergency Medicine, Vanderbilt University Medical Center; 13 Assistant Professor, Clinical Pediatrics, Division of Emergency Medicine, Medical College of Wisconsin; 14 Associate Professor, Clinical Pediatrics, Division of Emergency Medicine, University of Washington School of Medicine and Seattle Children's Hospital; 15 Assistant Professor, Clinical Pediatrics, Division of Emergency Medicine, University of Washington School of Medicine and Seattle Children's Hospital

**Keywords:** Neonate, Newborn, Infant, Simulation, Vitamin K Deficiency, Cerebral Hemorrhage, Homebirth, Critically Ill, Critical Illness, Neonatal-Perinatal Medicine, Pediatric Critical Care Medicine, Pediatric Emergency Medicine, Pediatrics

## Abstract

**Introduction:**

The American Academy of Pediatrics recommends vitamin K prophylaxis at birth for all newborns to prevent vitamin K deficiency bleeding (VKDB). Despite a lack of evidence for serious harms, barriers to prophylaxis, including parental refusal, are rising, as are cases of VKDB.

**Methods:**

This simulation involved an infant presenting to the emergency department who decompensated due to a cerebral hemorrhage caused by VKDB and was treated by pediatric and emergency providers. The case was incorporated into the fellow and division monthly curricula, and participants completed postsimulation surveys. The patient required a secure airway, seizure management, vitamin K, and a fresh frozen plasma infusion upon suspicion of the diagnosis, plus a coordinated transfer to definitive care. The case included a description of the simulated case, learning objectives, instructor notes, an example of the ideal flow of the scenario, anticipated management mistakes, and educational materials.

**Results:**

The simulations were carried out with 48 total participants, including 40 fellows and eight attendings, from five different training institutions over 1 year. In surveys, respondents gave overall positive feedback. Ninety-four percent of participants gave the highest score on a Likert scale indicating that the simulation was relevant, and over 80% gave the highest score indicating that the experience helped them with medical management.

**Discussion:**

This simulation trained physicians how to recognize and treat a distressed infant with VKDB. The case was perceived to be an effective learning tool for both fellow and attending physicians.

## Educational Objectives

By the end of this module, the learner will be able to:

1. Demonstrate an appropriate initial approach to a critically ill newborn, including stabilization of airway, breathing, and circulation.
2. Identify impending neurologic decompensation and coagulopathy and formulate a differential diagnosis in the setting of a home birth, including vitamin K deficiency.
3. Develop and execute a management plan for a patient with seizure and acute cerebral hemorrhage in the setting of vitamin K deficiency.
4. Demonstrate effective team leadership, roles, and communication.

## Introduction

Vitamin K deficiency bleeding (VKDB), previously known as hemorrhagic disease of the newborn, is the acquired coagulopathic state of infants attributable to the inability to activate vitamin K–dependent coagulation factors (II, VII, IX, and X). Manifestations of the disease include overt bleeding, vomiting, poor feeding, lethargy, and intracranial hemorrhage that may necessitate urgent neurosurgical intervention. The coagulopathy is rapidly corrected with administration of vitamin K and fresh frozen plasma (FFP). The American Academy of Pediatrics recommends vitamin K prophylaxis to prevent VKDB, an evidence-based intervention that has led to a decrease in major bleeding of infants.^1^ Despite the recommendation, there is a rise of this life-threatening coagulopathy in young infants due to lack of prophylaxis at birth.^2^ The media highlighted four cases of VKDB in Tennessee where infants who never received vitamin K developed sudden bleeding between 6 and 15 weeks of age.^3^ Three of these children developed intracranial hemorrhage, one with resultant gross motor deficits. Recent research has revealed multiple barriers to vitamin K adherence, including the debunked theory of cancer association and parental fears of pain and vaccinations, to name a few.^4^

Currently, there are no *MedEdPORTAL* publications on this specific topic. There are several simulation-based publications on topics regarding the ill neonate, including critical congenital heart disease,^5^ epilepsy,^6^ neonatal resuscitation,^7^ febrile infant,^8,9^ infant in shock,^10,11^ and nonaccidental trauma,^12^ as well as one resource addressing newborn medications that includes a discussion of vitamin K.^13^ Our simulation case can be seen, therefore, as part of this series on the critically ill neonate in an emergency setting.

In an effort to provide an effective learning activity on the topic of VKDB for the adult learner, we chose simulation as the educational strategy to facilitate an active learning environment through participation. The simulation was designed to be run with both high- and low-fidelity equipment. This educational method was selected to provide a risk-free learning experience dedicated to this critical, yet rare, clinical situation. The target audience of this simulation included trainees and attending physicians working in the emergency medicine setting who would be responsible for treating a sick infant. Given that this educational activity was integrated into routine and ongoing didactics with hospital employees, institutional review board approval was not obtained. This simulation can be viewed as one part of a larger series published in *MedEdPORTAL* by the current group of authors: the Pediatric Emergency Medicine Simulation Curriculum.^14–19^ We chose to include attending physicians from various disciplines in the simulation when available in an effort to more effectively emulate reality.

## Methods

### Development

We designed this resource to supplement any pediatric emergency medicine (PEM) simulation training program, acknowledging the lack of a prior published VKDB case in *MedEdPORTAL*. We constructed the case to teach the recognition and management of an ill and decompensating infant presenting to the emergency department (ED), including medical, skill, and teamwork/communication learning objectives. In order to facilitate an active learning environment, we designed the curriculum with a simulation scenario. Learners were expected to do a primary and secondary assessment according to the Pediatric Advance Life Support systemic approach algorithm^20^ and to recognize signs of altered mental status and coagulopathy risk in the patient. Learners were also expected to assign team roles, effectively communicate as a team and with the parent, recognize a sick infant and call for help, obtain a thorough patient history and identify the risk of VKDB, stabilize and treat the patient, and arrange transfer to the appropriate definitive care team. Cconsistent with other resources in the Pediatric Emergency Medicine Simulation Curriculum series published in *MedEdPORTAL,* we designed content to assist with preparation, setup, facilitation, and debriefing; a guide to teamwork and communication; and a postsimulation survey. More specific to this case were the simulation, critical actions checklist, relevant labs/imaging, standardized patient script, and reference materials including a short PowerPoint presentation and one-page educational handout. These educational materials could be reviewed by the learner prior to or after the simulation to review clinical content.

### Equipment/Environment

We conducted this simulation in the hospital ED resuscitation bay during designated simulation education time. Prior to the simulation, the facilitator and educational team prepared the room with the mannequin and bedside materials including monitors, IV/intraosseus infusion (IO) equipment, respiratory and intubation supplies, and a printout of labs/imaging. All of the centers that ran this simulation used high-fidelity mannequins (i.e., an operator controlled the physiologic parameters on the mannequin and corresponding monitor). Moulage was added to the mannequin in the form of makeup to depict scattered bruising. This being said, the case need not be limited by mannequin fidelity and could be adapted to low fidelity using a simple baby doll with moulage and a monitor application streamed on a tablet, with the facilitator providing the vital sign and physical exam changes verbally.

### Personnel

The personnel needed to successfully run this simulation included at least four people. The two who ran the simulation were a facilitator/debriefer and a standardized patient parent who also served as an embedded participant. The two who participated in the simulation were a nurse and a medical provider (this number can be adapted depending on who would realistically be available to respond to the patient at another institution). To prepare the simulated patient, the faculty met the actor prior to the simulation to review the scenario and discuss the learning objectives of the case. We wrote this case for formative educational use rather than for standardized testing; therefore, we elected to use a simulated patient actor to allow for greater flexibility and authenticity across the various hospital emergency rooms in which the scenario was run. We were liberal in our selection of patient actors, choosing available and interested personnel among our faculty and personal acquaintances. We recommend that future users of this resource refer to the Association of Standardized Patient Educators (ASPE) for the established best practices for the use of standardized patients.^21^

### Implementation

We implemented this 1-hour simulation over 1 year at five US academic children's hospitals with 48 total participants, including 39 PEM fellows, one neonatal intensive care unit (NICU) fellow, and eight PEM attendings. Each participant completed the exercise only once. The case was incorporated into the fellow simulation curricula at four of the sites and into the division monthly simulation curriculum at one site (where the attendings and NICU fellow were incorporated). Participant teams included at least four providers (provider 1: team lead; provider 2: airway/survey physician; provider 3: helper to perform interventions such as ordering medications, contacting specialist help, placing IO, etc.; provider 4: attended to parent, obtained history) and two bedside nurses assigned the roles of placing monitors, getting IV/IO supplies, and administering medication. On the occasion when no nurse was available, a physician played the role of the nurse. The simulation was run as an interdivision simulation at one institution, where the PEM fellows and attendings stabilized the patient and called the NICU fellow to the bedside. The other institutions ran this simulation as part of a monthly PEM fellows simulation curriculum, with participants limited to fellows and the team available to them, including registered nurses (RNs) and respiratory therapists (RTs). Some institutions had a pharmacist in attendance. All institutions used high-fidelity mannequins, where an operator managed the state of the mannequin and monitor, adapting the vitals and physiologic state to participant medical interventions. This case could be adapted to include the team members who would normally respond to a sick infant, as well as to run with a low-fidelity setup.

Prior to the simulation (Appendix A), the facilitators reviewed the environment preparation document (Appendix B) for specifics about the simulation setup, including a sample prebrief script and cognitive aids/resources, medications, and equipment to have available. Prior to the activity, the participants were prompted that this would be a simulation case but to act and physically do everything as if it were a real patient scenario. The scenario began with the standardized patient parent rushing in carrying the bruised and fussy baby. The triage nurse immediately recognized the infant as sick, brought the infant to the resuscitation room, and called for prompt help. Labs/imaging were available upon request (Appendix C) and made available to those institutions with the capacity to order such tests. The facilitator used the critical action checklist (Appendix D) to note the specific learning objectives of the case as they happened and to prompt the debrief. The facilitator/debriefer used the guides on the debrief (Appendix E) and teamwork/communication (Appendix F) to effectively lead the postsimulation discussion. The facilitator/debriefer was encouraged to reinforce the medical knowledge learning objectives with the prepared PowerPoint (Appendix G) and handout (Appendix H) aids during the postsimulation discussion. A script for the standardized patient was also provided (Appendix I). Finally, the participants were asked to complete the postsimulation survey (Appendix J) prior to being dismissed from the session.

### Assessment

Participants provided feedback on the evaluation form about whether the simulation was relevant, realistic, and performed in a safe learning environment. We developed the questionnaire to elicit participant reflection on whether the simulation was perceived to be effective in teaching basic skills, medical management, and teamwork. We asked participants to comment on how the simulation might alter their medical management in the future (Kirkpatrick level 2^22^). Finally, we encouraged the participants to remark on the simulation as a whole (Kirkpatrick level 1^22^). We did not make an attempt to measure knowledge before the simulation; future facilitators might decide to do so to evaluate the impact of the simulation on the knowledge acquisition of participants.

### Debriefing

We allowed approximately twice the amount of time used in the simulation for debriefing. The debriefing materials were adapted from our prior *MedEdPORTAL* publications.^16,18^ The PowerPoint slides and educational handout were provided to the participants for review both before and after the simulation. The primary facilitator, who also served as the debriefer, utilized the critical actions checklist to constructively organize the educational goals and feedback. This checklist was created in conjunction with PEM and NICU attending input as to what the priorities should be for managing this patient. The embedded participant could be invited to participate also. If they are a simulation-trained person, they can help facilitate the debriefing; if not, they can provide feedback from a patient perspective.

## Results

All 48 participants completed the postsimulation survey. We intentionally sought out participants from five different US children's hospitals in an effort to obtain feedback from a variety of educators and trainees.

Participants scored the survey questions using a 5-point Likert scale (1 = *strongly disagree,* 3 = *neutral,* 5 = *strongly agree*). When asked if the curriculum content was relevant to their work, 94% of participants answered with a 5 (median = 5, range = 4–5). When asked if the scenario was realistic, 77% answered with a 5 (median = 5, range = 3–5). When asked if the simulation was effective in teaching the recognition of VKDB, 85% answered with a 5 (median = 5, range = 4–5). When asked if they felt prepared to stabilize the patient, 71% answered with a 5 (median = 5, range = 3–5). When asked if the case was effective in teaching how to manage the patient, 81% answered with a 5 (median = 5, range = 3–5). When asked if they felt comfortable activating assistance early, 81% answered with a 5 (median = 5, range = 3–5). When asked if they felt able to practice teamwork and communication, 73% answered with a 5 (median = 5, range = 3–5). When asked if the debrief created a safe environment, 75% answered with a 5 (median = 5, range = 4–5). When asked if the debrief promoted reflection and team discussion, 90% answered with a 5 (median = 5, range = 4–5). These results are presented in the Table.

**Table.**
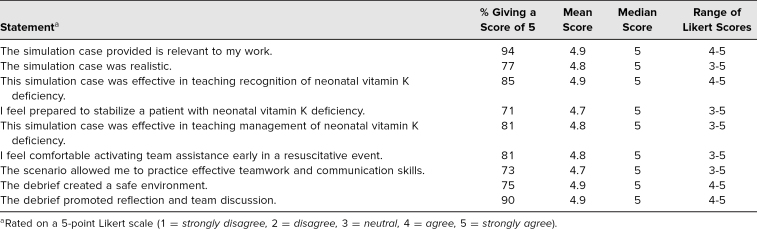
Results From Postsimulation Questionnaire

When asked how the simulated experience would change how learners approached their job, respondents overall agreed that they would take the time to obtain a thorough prenatal, birth, and past medical history of the sick and decompensating infant, including specifically asking about vitamin K given at birth and route given (intramuscular [IM] vs. by mouth). Several participants admitted to having an anchoring bias on the diagnosis of inflicted physical abuse on a bruised baby and appreciated the opportunity to discuss a broader differential including coagulopathy. Many learners did not realize vitamin K deficiency bleeding could be the etiology through 6 months of age, so would keep that in mind with this patient population. Several learners commented that the simulation helped them think through prioritization of interventions, given the relative complexity of the case. The fellow learners, in particular, overall expressed the importance of learning about the treatment of VKDB with vitamin K, with specifics about route of administration, even before the lab results returned if there was a concerning history. Similarly, they learned to order FFP and blood products when there was concern for VKDB and ongoing bleeding.

When asked how this scenario could be improved, several participants who participated in sessions with only physician providers, without nurses, reiterated the importance of recruiting an entire ED medical team including RNs, RTs, pharmacists, and whomever else would normally be present to participate. At one site, the monitor did not work, which diminished the realism of the activity. One group did not use moulage to paint the bruises on the mannequin, which made the case unrealistic for the participants. One NICU fellow reported that it would be nice to more consistently work together with the ED folks to ensure good thermoregulation, IV access, and difficult airway management. We would love to have more visibility as a resource to the ED so I think these types of events are great opportunities to build those lines of communication… (one way to improve the simulation) is to give the PEM team a heads up call from the ambulance and ensure they have enough time (or a prompt) to call the NICU before the baby arrives so we can have them practice how to incorporate ICU help into their code management.

## Discussion

We devised this resource as a drill to support pediatric and emergency providers in recognizing and treating an ill infant with VKDB. Given that the case was conducted in academic children's hospitals with robust simulation centers in the pre-COVID-19 era, we designed it for the high-resource, in-person setting. This being said, the learning objectives could certainly be met using low-fidelity simulation setups or even remotely via telesimulation. The mental exercise of how to approach an ill infant with this history and presenting physical exam, including teamwork/communication skills, discussion of the differential diagnosis, and verbalizing actions that one would do if the patient were present, is worthwhile. Therefore, we believe this simulation is adaptable for use across the simulation delivery spectrum.

We encountered challenges related to the feeling of reality of the simulated patient scenario when participants were limited to only physicians (without the more real interdisciplinary ED medical team including nursing, RT, and pharmacy in attendance), when the mannequin or monitor malfunctioned, and when the moulage of bruising was not obvious to the learner or not reiterated by the facilitator. We found that incorporating a simulated patient to play the patient's mother—selected from among the medical faculty—was an effective way to heighten realism. Should this case be used for formative testing, we recommend utilizing standardized patients as defined by the ASPE.^21^ Additionally, future groups might consider incorporating the actor's feedback into the debrief to highlight the patient's perspective.

As for learning objectives, across all learners (fellows and attendings alike) we found that the most important piece was discovering the vitamin K deficiency on history, recognizing the risk for a cerebral hemorrhage in this neurologically impaired patient with bruising, and promptly calling the NICU and neurosurgical teams to help as soon as possible. We reiterate the importance of ordering and administering vitamin K via IM and FFP via IV as soon as possible upon clinical suspicion, rather than waiting for results of coagulopathy on labs or head CT, which could take a while to obtain, if not being impossible altogether. Given the complexity of a case requiring IO access, intubation, seizure management, consideration of a broad differential diagnosis, and a rare diagnosis, we found that focusing the discussion on the prioritization of interventions as made clear by the team leaders was useful for the participants. It was helpful to consolidate the medical and teamwork/communication learning objective takeaways in the debrief, utilizing the provided education materials (PowerPoint and handout) following the simulation. Should educators consider using this case with novice learners (e.g., medical students, residents), they might consider distributing these educational materials prior to the simulation.

Our general recommendation to facilitators is to let the participants get as far as they can in the time allotted for the simulation with minimal outside nudging or assistance, then utilize the debrief to learn from what went well and what could be improved next time. For example, if nobody asks the parent for a birth history and therefore never discovers the lack of Vitamin K prophylaxis, that is a major takeaway for the debrief discussion.

Respondents gave overall positive feedback on the Likert scale and wrote comments supporting the case as an effective learning tool for both fellow- and attending-level learners. However, we limited the postsimulation survey evaluation to learner perceptions of utility and did not evaluate impact on the knowledge, acquisition, or communication learning objectives. In the future, one might consider giving pre- and posttests consistent with the specific case learning objectives, conducting iterative simulations and capturing adherence to and timeliness of critical actions (Appendix D), and/or videotaping with subsequent review, all of which are ways to raise the evaluation to higher Kirkpatrick levels.^22^

## Appendices

VKDB Simulation Case.docxVKDB Sim Environment Preparation for Facilitator.docxVKDB Labs Imaging.docxVKDB Critical Action Checklist.docxVKDB Debrief.docxVKDB TeamSTEPPS.docxVKDB Didactic PowerPoint.pptxVKDB Handout.docxVKDB Standardized Patient Script.docxVKDB Postsim Survey.docx
All appendices are peer reviewed as integral parts of the Original Publication.
