# Supplementary material for: Pediatric Emergency Medicine Simulation Curriculum: Vitamin K Deficiency in the Newborn
Source: MedEdPORTAL. 2021 Jan 25;17:11078. doi: 10.15766/mep_2374-8265.11078 (PMC7830750; doi:10.15766/mep_2374-8265.11078)
Supplement: Supplementary file 1 — VKDB Simulation Case.docxVKDB Sim Environment Preparation for Facilitator.docxVKDB Labs Imaging.docxVKDB Critical Action Checklist.docxVKDB Debrief.docxVKDB TeamSTEPPS.docxVKDB Didactic PowerPoint.pptxVKDB Handout.docxVKDB Standardized Patient Script.docxVKDB Postsim Survey.docx [file mep_2374-8265.11078-s001.zip › A. VKDB Simulation Case.docx]

| **Appendix A: MedEdPORTAL Simulation Case Template**  **SIMULATION CASE TITLE:** Pediatric Emergency Medicine Simulation Curriculum: Vitamin K Deficiency in the Newborn  AUTHORS: Elizabeth Sanseau, MD, MS, Leah H. Carr, MD, Jennifer Case, MD, Khoon-Yen Tay, MD, Anne Ades, MD, Kesi Yang, MD, Hannah Huang, PharmD, Anna Bustin, PharmD, Grace Good, MA, Shannon Gaines, RN, MSN, Julie Augenstein, MD, Daisy Ciener, MD, MS, Jean Pearce, MD, MS, Jennifer Reid, MD, Kimberly Stone, MD, MS, Rebekah Burns, MD, Anita Thomas, MD, MPH | |
| --- | --- |
| **PATIENT NAME:** Hallie  **PATIENT AGE:** 4-week-old  **CHIEF COMPLAINT:** irritability, lethargy, tremulousness | |
|  | |
| **Brief narrative description of case** | You are called to the resuscitation bay of your Emergency Department after an infant was rushed through triage by a nurse. In triage, the nurse recognized the baby appeared irritable and unwell in her mother’s arms. The mother is visibly upset, crying and asking if her baby will be okay.  The anticipated interventions of the responders include: (1) surveying the patient according to the Pediatric Advanced Life Support (PALS) algorithm: initial impression, primary assessment, secondary assessment; (2) recognizing and initiating treatment for neurologic illness in an infant (i.e. irritability, lethargy, signs concerning for anemia such as tachycardia, signs concerning for coagulopathy or trauma (bruising), irregular movements concerning for seizure); (3) recognizing and initiating treatment for potential vitamin K deficiency bleeding (i.e. history of home birth, no vitamin K given, bruising on exam); and (4) activating NICU support and neurosurgical consultation (if available) to aid in further stabilization and management.  Anticipated interventions include management and further evaluation of neurologic irregularities (e.g. discussion of available head imaging (CT, head ultrasound [US]), potential need for electroencephalography [EEG]) recognition of need for intravenous (IV) access and use of intraosseus (IO) access when IV management is unsuccessful, securement of an airway via endotracheal intubation, recognition and treatment of bleeding diathesis from history and management with vitamin K and fresh frozen plasma (FFP), treatment for presumed sepsis with broad spectrum antibiotic and antiviral medication, and involvement of the neonatal intensive care unit (NICU) and consideration of neurology and neurosurgical team involvement (as is available at your institution).  The patient stabilizes after obtaining access, intubation, antiepileptics, fluid bolus, FFP and vitamin K administration with broad-spectrum antibiotics and antivirals. |
| **Primary Learning Objectives** | Medical Management Learning Objectives:   1. Demonstrate an appropriate initial approach to a critically ill newborn, including stabilization of airway, breathing and circulation. 2. Completion of a primary and secondary survey. 3. Secure IO access after failing IV x 3. 4. Recognize and treat status epilepticus. 5. Consider neonatal sepsis diagnosis and discuss treating with fluids and broad-spectrum antibiotic and antiviral medications. 6. Perform time-out and secure an airway via endotracheal intubation. 7. Obtain the history of a homebirth without Vitamin K administration, leading to the coagulopathic state. 8. Activate the NICU and consider involvement of neurology and neurosurgical teams (as is available at your institution). 9. Order and administer Vitamin K and FFP as soon as recognize risk. 10. Recognize cerebral hemorrhage on non-contrast head CT vs US (if available).   Team Management Learning Objectives:   1. Clear roles and responsibilities    - Well-defined roles and responsibilities, including identification of a clear team leader 2. Situational awareness 3. Directed, closed-loop communication |
| **Ideal Scenario Flow** | The learners walk in to the resuscitation bay and approach the distressed mother and patient. One learner addresses the parent and brings them away from the bedside so the patient can be assessed by the team. They assign team roles (including team leader, airway/survey doc, caller for help, and provider of patient care activities). They perform an evaluation, including an initial impression, primary and secondary assessment, and continue to reassess the patient. They acknowledge the abnormal vital signs seen on the monitor or reported by the bedside nurse (patient should be initially tachycardic) along with abnormal physical exam findings (i.e. irritability, lethargy, bruising on exam) before decompensating in the ER bay. The provider asks historical questions of the patient’s mother and is able to glean that the patient was born at home and did not receive vitamin K. This information is effectively conveyed to the team at the bedside. The learners consult the NICU for additional assistance as soon as possible. The ED team works in conjunction with NICU learners and initiates plans to gain IV access and successfully places an IO when they are unable to place an IV. They then perform a timeout and secure the patient’s airway and discuss the need for anti-seizure medications, treatment for possible sepsis with fluids and broad spectrum antibiotics/antivirals, labs (including coagulation studies) and head imaging, and neurosurgical consultation. Given the recognized risk for potential intracranial hemorrhage, they order and administer vitamin K and FFP. The patient stabilizes and is transferred to the NICU for additional management. |
| **Critical Actions** | See **Appendix C**. |
| **Learner Preparation** | - PALS systemic approach handout (can be downloaded from the American Heart Association) - PowerPoint presentation and Handout covering basic learning objectives for vitamin K deficiency in the newborn (Appendix F, G) - Reference to treatment of VKDB in a neonate such as F&L (https://pediatrics.aappublications.org/content/112/1/191) - Teamwork and communication glossary [Appendix E; TeamSTEPPS: national implementation. Agency for Healthcare Research and Quality Web site. http://teamstepps.ahrq.gov. Accessed February 20, 2018.] |

| Initial Presentation | |
| --- | --- |
| **Initial vital signs** | HR: 180 BP: 95/60 (MAP 72) RR: 40 Temp: 37deg C SpO2: 90% RA Wt: 4kg  Behavior: Irritable and intermittently not responsive to examination  Skin: cool, several bruises seen over extremities |
| **Overall Appearance** | Hallie is lying in her mother’s arms, irritable, and appears difficult to console. She does not open her eyes spontaneously. |
| **Actors and roles in the room throughout case** | Group of 4 provider team members (medical students, residents, fellows or attendings) enter into the resuscitation bay with 2 bedside nurses, eventually 1-2 NICU providers (fellows, attendings) join with 1-2 NICU nurses and a respiratory therapist:  Provider #1: Team lead  Provider #2: Airway/survey physician  Provider #3: Helper who performs interventions, such as orders medications, contacts consultants, places IO  Provider #4: Gains history and calms patient’s parent  ED bedside RN #1: Wait to be assigned role by team lead, should assist with medication administration  ED bedside RN #2: Wait to be assigned role by team lead, may assess vitals, placing monitors and assist RN #1  Simulated patient: High or low-fidelity neonatal manikin  Simulated patients’ parent: Played by a standardized patient (SP) or actor (see Appendix H)  NICU provider #1 and #2: Wait to be assigned role by team lead, may assist with management decisions and interventions  NICU RN #1 and #2: Wait to be assigned role by team lead, may assisted ED RNs  Respiratory therapist: Wait to be assigned role by team lead, may assist with intubation and transition to ventilator  Instructor: Simulation instructor who will also lead debriefing. |
| **HPI** | Instructor volunteers vignette:  Hallie is a 4-week-old (4kg) infant who is brought to the ED by her mother who reports that over the last few days she has become increasingly fussy and is not waking to breastfeed like she had immediately after birth. She has also had several episodes where she seems to be a little shaky. The nurse calls the team because the baby appears “lethargic” and has diffuse bruising on her trunk and bilateral upper and lower extremities.  Simulated patient’s parent, when asked about medical history:  *Birth history:* Hallie had an uneventful, non-traumatic home birth with her father and a naturopath in attendance. She was born at 39 weeks gestation. She went immediately to breast following birth and required no resuscitation. She did not receive erythromycin ointment, Hepatitis B or a vitamin K injection at birth. Normal prenatal history, Mother took no prenatal medications. No known family history of vascular, hematologic, biliary neurologic disease or seizures.  *Medical history:* She has been seen regularly by her pediatrician and has been growing well with exclusive breastfeeding. Her newborn screen returned normal. Her doctor has not had any concerns. She was seen by the Naturopath for some bleeding at the umbilical stump at 1 week of life, but was told that it was normal and no workup was obtained. She was reportedly well up until about 5 days ago when she was noted to be sleepy. Irritability, intermittent lethargy, and tremulouslessness have worsened since then. No fevers or localizing infectious symptoms.  *Family history* *and maternal prenatal labs:* First infant to these parents. Family medical history is unremarkable. If asked specifically about bleeding or neurologic or seziure disorders, there is no known family history.  *Social:* Mother, father, and grandparents are the only individuals who have cared for the baby. If asked specifically, there have been no social concerns. No concern for non-accidental or accidental trauma. |
| **Physical Examination** (initial impression) (primary assessment - abnormal in red) | |
| **General** | HR: 180 BP: 95/60 (MAP 72) RR: 40 Temp: 37deg C SpO2: 90% RA Wt: 4kg  Held in mother’s arms, initially appears irritable despite the mother trying to rock the infant.  When placed on the exam table, becomes lethargic and quiet.  Does not spontaneously open her eyes.  Grossly non-dysmorphic. |
| **HEENT** | Patent airway, mucous membranes appear dry.  No obvious trauma to head. No facial/head/ear bruising. No subconjunctival hemorrhage or icterus.  Pupils sluggish, but equally reactive to light (*if have pen light to assess).*  Fontanelle bulging and hard. |
| **Neck** | Supple. |
| **Lungs** | RR 40-60 breaths per minute.  Normal lung sounds bilaterally.  SpO2 90% RA. |
| **Cardiovascular** | HR 180.  BP 95/60 (MAP 72). *(if asked - 4 extremity BPs without marked difference)*.  Brachial and femoral pulses symmetric, but weak.  Capillary refill >3 seconds.  Mottled arms and legs. |
| **Abdomen** | Soft, non-tender, non-distended.  No hepatosplenomegaly.  Patent anus, no hair tuft or sacral dimple. |
| **Neurological** | Intermittently irritable and lethargic, intermittent tremors.  Intact and symmetric Moro. Intermittent intact suck and rooting reflex. |
| **Skin** | Pale, cool to touch.  Scattered bruising without recognizable pattern noted over bilateral upper and lower extremities and throughout trunk (chest and back).  No evidence of burn or fracture.  No petechiae. |
| **Musculoskeletal** | Normal bilateral hip exam. No effusions or obvious dislocations noted at joints. No obvious bony fractures or dislocations. |

| Instructor Notes - Changes and CASE Branch Points | | | |
| --- | --- | --- | --- |
| **Intervention / Time point** | **Change in Case** | **Additional Information** |  |
| Providers approach patient and patient’s mother. |  | Patient’s mother is distraught, asking if “her baby is going to be okay?” |  |
| One provider approaches patient’s mother, helps calm her, and encourages her to place patient on the examination table and step to the side of the bed. |  | Patient’s mother calms and continues to ask questions in a less-distraught manner. |  |
| Team lead divides up learner roles:  Provider #1: Team lead  Provider #2: Airway/survey   physician  Provider #3: Helper who performs   interventions  Provider #4: Gains history and   calms patient’s parent.  ED bedside RN #1: Assist with   medication administration  ED bedside RN #2: Assess vitals   and assist RN #1 | Patient is fussy, inconsolable, and becoming more lethargic.  Has bulging fontanelle.  Pupils are sluggish, but equally reactive to light. |  |  |
| Participants complete PALS initial assessment, primary and secondary assessment.  Acknowledge that the patient has bruising with an abnormal neurological and respiratory examination. | Patient is irritable and intermittently becomes unresponsive with sustained shaking movements (no longer intermittent) with associated apnea. | Patient’s mother says, “You don’t think my baby’s seizing, do you?” |  |
| Team recognizes neurologic and respiratory decompensation and calls a code.  Start CPAP.  Start bagging. | Infant apneic on CPAP, providers must bag infant as prepare for intubation.  Successful ventilation and oxygenation with bagging. | Patient’s mother is upset, but calmed easily if updated throughout this process. |  |
| Team recognizes status epilepticus and recognize the need for airway management, IV access, and antiepileptic medication.  Three attempts at an IV are unsuccessful, IO placement successful, labs are ordered. | Seizure like movements not inhibited by IV/IO attempts, non-suppressible movements. |  |  |
| Once IO access obtained, airway physician with team leader coordinate a time out and plan for antiseizure medications and intubation. |  | Labs “pending.” |  |
| Seizure medications ordered (or whatever you use at your institution):   - Ativan 0.05 mg/kg administered over 2-5 mins to be repeated q10mins - Phenobarbital 20 mg/kg | Seizure stops after one dose of Ativan. | Ativan is provided immediately. Phenobarbital is “pending from pharmacy.” |  |
| Team takes a timeout and decides to intubate.  RSI medications:   - Possible pre-med: Atropine 0.02 mg/kg (to blunt vagal response during the procedure) - Sedative for emergent intubation (e.g. fentanyl 1-2 mcg/kg/dose) - Paralytic medication, per institution (e.g. vecuronium 0.1 mg/kg)   Intubate with a 3.5 uncuffed ETT using 0-1 laryngoscope blade to a dept of ~9 cm at the lip in coordination with medications given: +/-pre-med, sedative, paralytic.  Order CXR to confirm placement. | Intubation is successful with or without premedication (*depending on what is the normal at your institution*).  SpO2 100%, RR 40-60 on ventilator, good waveform on capnography, purple to yellow change on colorimeter, ETCO2 35, good chest rise and symmetry breath sounds, skin pinking up. | CXR is provided - confirms appropriate ETT placement. |  |
| Team considers neonatal sepsis, orders fluids and broad spectrum antibiotic and antiviral medications.  Sepsis fluids and medications:   - NS bolus: 10 cc/kg - Ampicillin: 75 mg/kg - Cefotaxime: 50 mg/kg - Acyclovir: 20 mg/kg   Reassess head to toe patient following first fluid bolus. | Following NS bolus of 10cc/kg, skin more pink, capillary refill 2-3 seconds. No crackles or enlarged liver edge.  Pupils are sluggish, but equally reactive to light. | Pharmacy delivers medications. |  |
| Team recognizes need for help including head imaging and neurosurgery and NICU consultation. |  | Neurosurgery suggests getting head CT and to call back once it has arrived.  NICU is “en route.” |  |
| Provider learns about patient’s history of homebirth and lack of vitamin K administration.  Asks about risk/known history of trauma. |  | Patient’s mother says, “We didn’t think the shots at birth were necessary so we refused and she was doing fine.”  “My baby has never left my side. I did not hurt my baby – is that what you’re asking?!” |  |
| Given concern for Vitamin K deficiency bleeding, while coagulation studies are still pending, team orders treatment:   - Vitamin K (2mg IM) - FFP (10-15 mg/kg IO) |  | Pharmacy delivers medications. |  |
| Team calls/greets NICU:  NICU team’s assigned roles:  NICU provider #1 and #2: may   assist with management decisions   and interventions.  NICU RN #1 and #2: may assisted   ED RNs.  Respiratory therapist: may assist   with intubation and transition to   ventilator. |  | NICU team arrives and roles are assigned.  Patient’s mother asks, “Who are these people?!” |  |
| Team works with NICU to optimize thermoregulation, discuss broad differential and next steps while await labs and prep to bring to CT scan.  Team asks if labs have returned.  Upon obtain labs, recognize a coagulopathic state consistent with vitamin k deficiency. |  | Labs return (Appendix I): (abnormal in red):  WBC 8 (diff pending)  Hgb 6.5 (Hct 15)  Plt 140  PT >30 seconds (normal 10.0-14.3)  INR 3 (normal 0.53-1.26)  aPTT 100 seconds (normal 32.0-55.2)  Fibrinogen 2.7 (normal 1.62-3.78)  Venous iSTAT  7.01/60/-8, lactate 4  Na 148  K 4.7  Glu 150 |  |
| Decision to transport to NICU and then to CT scanner when more stable.  Call neurosurgery. |  | If it is reasonable to obtain the CT head at your institution (Appendix I): subdural bleed.  Neurosurgery recommends giving FFP in preparation for surgery (if has not already been given following vitamin K.  Scenario ends. |  |

**Anticipated Management Mistakes**

The most common and consequential participant errors are listed here with a corresponding discussion of corrective measures. If the group is not meeting the learning objectives, we recommend transitioning into the role of an embedded nurse participant to nudge as necessary.

- Failure to assess patient with primary and secondary survey. If run with the novice learner, they might not be familiar with how to approach the ill patient via the primary and secondary survey. We suggest reviewing PALS and the initial approach to the sick patient prior to running this sim with novice learners. Otherwise, as the embedded RN participant can ask the learning what they’re assessment for and note their assessment that the baby is having difficulty breathing and is profoundly lethargic.
- Failure to recognize and evaluate the decompensating neurologic exam. As the simulated nurse embedded participant you may note that the patient looks dusky, mental status is declining, or point out a bruise, for example.
- Failure to involve the NICU: The facilitator can end the scenario by saying they are the NICU arriving to help, ask for sign out. This can be a debriefing point in the discussion.
- Failure to obtain IO access after failing IV attempts: As the embedded RN participant you can push the learners to think of ways other than IV to get critical fluids and medications into the decompensating patient.
- Failure to secure the patient’s airway: If participants do not intubate, it is OK to have them successful bag mask ventilate the baby until help arrives. If not supporting breaths, the infant decompensates when seizing into a respiratory collapse and the scenario will end.
- Failure to obtain birth history from parent: If nobody is seeking the medical history from the standardized patient (SP), nudge the SP to be assertive and ask what the team is doing for her baby to encourage parental interaction. This may prompt them asking the birth history and get at the Vitamin K deficiency piece.
