# Supplementary material for: Pediatric Emergency Medicine Simulation Curriculum: Vitamin K Deficiency in the Newborn
Source: MedEdPORTAL. 2021 Jan 25;17:11078. doi: 10.15766/mep_2374-8265.11078 (PMC7830750; doi:10.15766/mep_2374-8265.11078)
Supplement: Supplementary file 1 — VKDB Simulation Case.docxVKDB Sim Environment Preparation for Facilitator.docxVKDB Labs Imaging.docxVKDB Critical Action Checklist.docxVKDB Debrief.docxVKDB TeamSTEPPS.docxVKDB Didactic PowerPoint.pptxVKDB Handout.docxVKDB Standardized Patient Script.docxVKDB Postsim Survey.docx [file mep_2374-8265.11078-s001.zip › B. VKDB Sim Environment Preparation for Facilitator.docx]

Before starting to use simulation at your place of work, a few things need to be done to prepare. If your place of work already has this in place, you are set to go. If not, this will be your pre-work:

- Identify roles and responsibilities in case of a patient emergency (e.g., front desk staff calls 911, nursing assistant brings the emergency backpack/supplies)
- Identify what emergency supplies are needed and where they are located
- Consider co-locating your supplies and having items clearly identified

As you initiate your simulations, it’ll be important for your work teams to have a shared mental model about the goals of the simulations, who is participating in the simulations, how often are the simulations, for how long and when they are taking place. Another thing to consider is if the sessions are scheduled or surprises. Here are a few thoughts to get you started:

- Consider goals in the following 3 categories:
  - Knowledge and skills: to increase learning around emergency management for a specific condition or situation (e.g., seizing infant)
  - Teamwork and communication: to increase effective teamwork and communication during an emergency (e.g., check backs, closed loop communication, situational awareness and roles/responsibilities)
  - Systems and processes: practicing in your own environment with your processes often identifies systems and process opportunities (e.g., clearer identification of emergency equipment location, missing equipment)
- How long and when:
  - Depending on your goals and frequency, allow enough time for doing the simulation and debriefing (talking about it afterwards). General rule of thumb is to allow 2x as long for debrief as action. So if you think your scenario is going to take 5 min, leave at least 10 min for debriefing and give yourself 5 min to get everyone on the same page = 20 min minimum. You may need more time at the start as people get used to the simulations.
  - Pick a time that is best for your work team. Perhaps a monthly meeting or before or after a scheduled workday. Consider setting aside the last patient slot of the day once a month/every other month/once a year for training.
- Scheduled vs surprise simulation:
  - Pros and cons to both. Consider the needs of your work teams. If simulation is something very new to your teams consider starting with scheduled and move towards surprise if the surprise element is important to your group.

For the actual simulations here are some tricks to get you started:

- As everyone gathers to start the session, take a few minutes to set the stage for a successful session:
  - Identify yourself and your role
  - Consider having everyone introduce themselves and state their role if the teams may not know each other so well
  - Let everyone know the plan for the session. E.g., doing one scenario and then debrief it.
  - Set the ground rules for the session:
    - Is each participant participating in their actual role?
    - Expectation for “really doing things”, e.g., if you need certain equipment, please go and get it. Clarify if you want participants to open closed packages, use real equipment, etc. or if you will be supplying some practice equipment.
    - We like to enter into a learning contract and invite our participants to join in order to create a safe learning environment. An example,

*“Whenever I do a simulation I enter into a learning contract with you and today I am asking you to join me in that learning contract in order to create a safe learning environment. The purpose of today’s session is to promote each other’s learning and to practice working together as a team. I make a basic assumption that everyone is intelligent, well-trained and doing their best. I am asking that today you suspend disbelief, doing the best you can to act as you normally would do so that we all may learn. I ask that you take away from here the learnings from the simulation but keep confidential the specifics of what takes place. This would not feel like a safe learning environment if afterwards you were to overhear someone saying, ‘I cannot believe what [*insert your name*] did during that simulation!’ All in agreement? Any questions? Let’s get started!*

During the simulation:

- If you are facilitating, let the action happen as much as possible without interruptions. If participants don’t know what to do, encourage them to think about and do what they might do in a real situation. Encourage participants to look up references that would be available in their unique pediatric practice. These are opportunities to discuss in the debrief.

For the Debrief:

- Provide enough time and a place to discuss. Consider if it’s the same space or separate space for the discussion. If you have a room where everyone can sit down and face each other that’s optimal (e.g., perhaps your waiting room).
- Quick debrief guide:
  - Two things that went well
  - Two things that didn’t go as well as we’d like
  - What could we do differently next time
- Try to end on a positive note

[Pediatric Emergency Medicine for Urgent Care Conference, March 2015, Seattle Children’s Hospital. Prepared by Drs. Kim Stone, Jennifer Reid and Rebekah Burns. Re-produced here with permission by authors.]

Before each simulation, ensure the anticipated resuscitation equipment is available for the team’s use.

**Resources**

Pediatric Resuscitation Medication references (e.g.: Broselow tape, reference cards)

Documentation forms

**Universal Precautions**

Gowns

Gloves

Mask and face shields

**Suggested Medications (consider having all or only a limited number of medications available – *have available only what your setting typically stocks*)**

| **Antibiotics / Antivirals:** | **Dose concentrations** |
| --- | --- |
| Cefotaxime | 50 mg/kg IV |
| Ampicillin | 75 mg/kg IV |
| Acyclovir | 20 mg/kg IV |
|  |  |
| **Antipyretics:** | **Dose concentrations** |
| Acetaminophen | 15 mg/kg PO |
| Ibuprofen | 10 mg/kg PO |
|  |  |
| **Fluids:** | **Dose concentrations** |
| Normal Saline (warmed or room temperature) | 20 mL/kg IV for standard bolus |
| Lactated Ringers (warmed or room temperature) | 20 mL/kg IV for standard bolus |
|  |  |
| **Anti-epileptics:** | **Dose concentrations** |
| Diazepam | 0.5 mg/kg PR (2-5yr), 0.3 mg/kg (6-11yr), 0.2mg/kg (>12yr) (max 10mg) |
| Lorazepam | 0.01-0.1 mg/kg IV for status epilepticus (max 4mg) |
| Midazolam | 0.2 mg/kg IN (1/2 in each nostril) (max 10mg) |
| Levetiracetam | 20 mg/kg IV for standard loading dose |
| Fosphenytoin | 20 mg/kg IV for standard loading dose |
| Phenobarbital | 15-20 mg/kg IV for standard loading dose |
|  |  |
| **Resuscitation and RSI:** | **Dose concentrations** |
| Atropine | 0.02 mg/kg IV |
| Epinephrine | 0.01 mg/kg (1:10,000) IV |
| Etomidate | 0.2 to 0.4 mg/kg IV |
| Fentanyl | 1-2 mcg/kg IV |
| Ketamine | 0.5-2 mg/kg IV |
| Propofol | 1.5-3 mg/kg IV |
| Rocuronium | 1 mg/kg IV |
| Vecuronium | 0.2 mg/kg IV |
|  |  |
| **VKDB reversals:** | **Dose concentrations** |
| FFP | 10-15 mg/kg IV |
| Vitamin K | 2mg IM |

**Equipment (consider having all or only a limited number of equipment available – *have available only what your clinic typically stocks*)**

Simulator in hospital gown or clothing, diaper, on bed with patient identification band

Moulage – Makeup for scattered bruising

Monitor – NIBP, HR, RR, Oxygen saturation, temperature and ETCO2 monitor (if available)

Blood Pressure cuff, Heart Rate monitor leads, Oxygen saturation probe, defibrillator cables and ETCO2 cannula (if available)

Oxygen hook-up on wall or cylinder

Shoulder roll

Bag-mask system, multiple size masks

O2 – nasal cannula, mask - simple and/or non-rebreather

Suction

Thermometer, temperature probe

Nasal, oral airways, multiple sizes

Endotracheal tubes- 3.0, 3.5, 4.0, 4.5, 5.0, cuffed or uncuffed, stylets

Laryngoscope, Miller and Mac blades, multiple sizes w/video assist (if available)

Laryngeal mask airway (LMA), multiple sizes

End-tidal CO2 colorimeter

Nasogastric/orogastric tube(s)

Stethoscopes

IV/Angiocath, various sizes

IO needles, 2 sizes

Gauze, Tape

IV tubing/blood product tubing and filters

IV pumps, pressure bags/ blood product pumps

Syringes, multiple sizes

Bedside blood sample processors: glucose, electrolytes, gases

Specimen tubes

Crash cart & backboard

Defibrillator / AED

High flow nasal cannula system and/or nasal CPAP system
