# Supplementary material for: Pediatric Emergency Medicine Simulation Curriculum: Vitamin K Deficiency in the Newborn
Source: MedEdPORTAL. 2021 Jan 25;17:11078. doi: 10.15766/mep_2374-8265.11078 (PMC7830750; doi:10.15766/mep_2374-8265.11078)

**Labs** (abnormal in red):

WBC 8 (diff pending)

Hgb 6.5 (Hct 15)

Plt 140

PT >30 seconds (normal 10.0-14.3)

INR 3 (normal 0.53-1.26)

aPTT 100 seconds (normal 32.0-55.2)

Fibrinogen 2.7 (normal 1.62-3.78)

Venous iSTAT

7.01/60/-8, lactate 4

Na 148

K 4.7

Glu 150

Type and cross: *pending*

**CXR:**

Author owned image


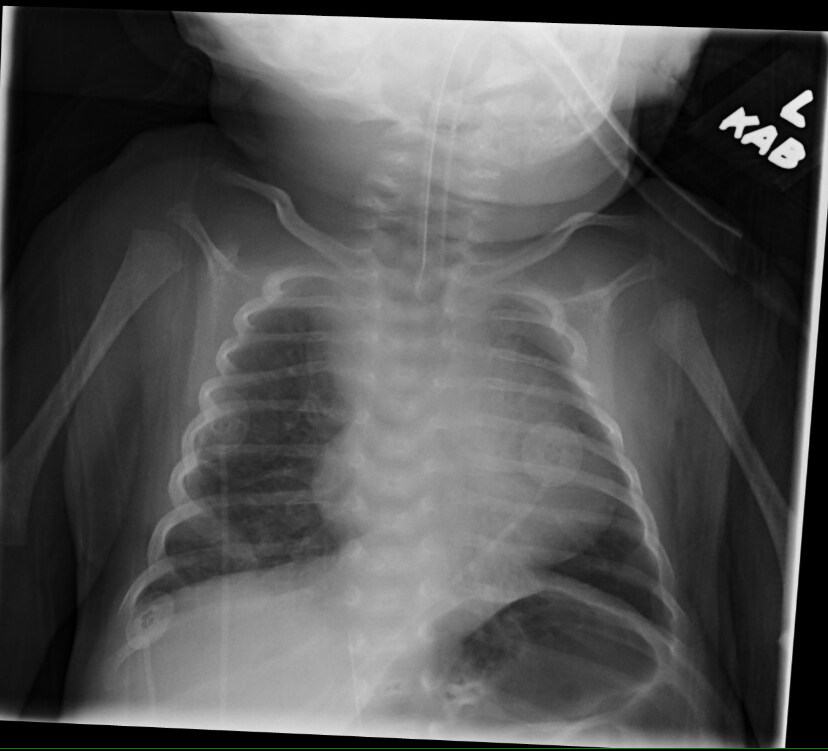


**Head CT:**

Author owned image


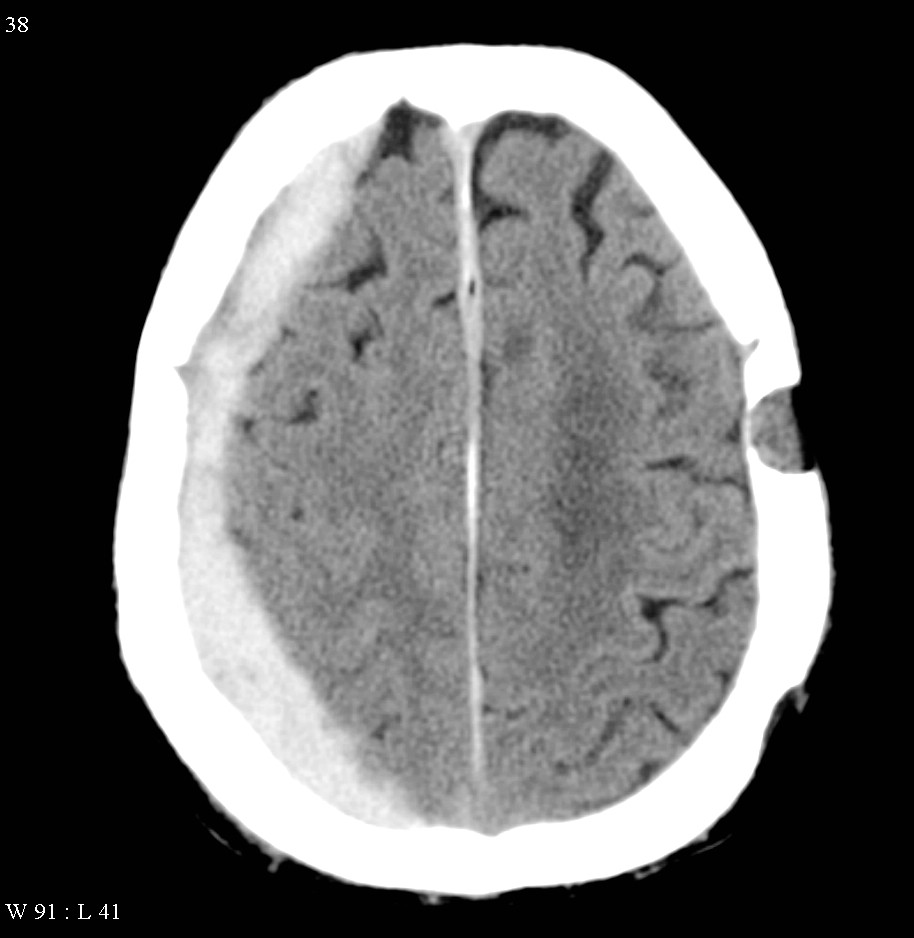

Supplement: Supplementary file 1 — VKDB Simulation Case.docxVKDB Sim Environment Preparation for Facilitator.docxVKDB Labs Imaging.docxVKDB Critical Action Checklist.docxVKDB Debrief.docxVKDB TeamSTEPPS.docxVKDB Didactic PowerPoint.pptxVKDB Handout.docxVKDB Standardized Patient Script.docxVKDB Postsim Survey.docx [file mep_2374-8265.11078-s001.zip › C. VKDB Labs Imaging.docx]
