# Supplementary material for: Pediatric Emergency Medicine Simulation Curriculum: Vitamin K Deficiency in the Newborn
Source: MedEdPORTAL. 2021 Jan 25;17:11078. doi: 10.15766/mep_2374-8265.11078 (PMC7830750; doi:10.15766/mep_2374-8265.11078)
Supplement: Supplementary file 1 — VKDB Simulation Case.docxVKDB Sim Environment Preparation for Facilitator.docxVKDB Labs Imaging.docxVKDB Critical Action Checklist.docxVKDB Debrief.docxVKDB TeamSTEPPS.docxVKDB Didactic PowerPoint.pptxVKDB Handout.docxVKDB Standardized Patient Script.docxVKDB Postsim Survey.docx [file mep_2374-8265.11078-s001.zip › D. VKDB Critical Action Checklist.docx]

| **Critical Action** | **Completed?** | **Notes** |
| --- | --- | --- |
| Demonstrate an appropriate initial approach to a critically ill newborn via primary survey (ABCDE) | Y/N |  |
| Completion of a secondary survey | Y/N |  |
| Secure an airway via endotracheal intubation | Y/N |  |
| Secure intraosseous access after failing intravenous access x 3 | Y/N |  |
| Appropriately manage seizure with antiepileptic drugs | Y/N |  |
| Formulate a differential diagnosis, including sepsis, seizure or metabolic disorder, intracranial mass or bleed, inflicted or accidental trauma, acquired/congenital bleeding disorder including vitamin K deficiency | Y/N |  |
| Recognize and treat coagulopathy with vitamin K and FFP (fresh frozen plasma) | Y/N |  |
| Recognize cerebral hemorrhage on head imaging (if available) | Y/N |  |
| Activate the neonatal intensive care unit and neurosurgical teams for further management (if available) | Y/N |  |
| Demonstrate effective team leadership, roles, and communication | Y/N |  |
