# Supplementary material for: Pediatric Emergency Medicine Simulation Curriculum: Vitamin K Deficiency in the Newborn
Source: MedEdPORTAL. 2021 Jan 25;17:11078. doi: 10.15766/mep_2374-8265.11078 (PMC7830750; doi:10.15766/mep_2374-8265.11078)
Supplement: Supplementary file 1 — VKDB Simulation Case.docxVKDB Sim Environment Preparation for Facilitator.docxVKDB Labs Imaging.docxVKDB Critical Action Checklist.docxVKDB Debrief.docxVKDB TeamSTEPPS.docxVKDB Didactic PowerPoint.pptxVKDB Handout.docxVKDB Standardized Patient Script.docxVKDB Postsim Survey.docx [file mep_2374-8265.11078-s001.zip › E. VKDB Debrief.docx]

**Debriefing Materials^[[1]](#footnote-1)^**

Simulation creates the opportunity to debrief. We believe that the focus of each simulation should be the DEBRIEF. Simulation creates the opportunity to examine our medical management, technical skills and teamwork and communication skills. It facilitates discussion about challenges in a safe environment in order to improve the quality of patient care.

Framework for debriefing

Each debrief should consist of 4 components:

- Introduction
- Discussion of emotions
- Discussion of medical management and technical skills
- Discussion of teamwork and communication skills

There is often overlap between medical management and teamwork issues. Debriefing may not follow a linear progression of all four of these components.

General Debriefing Goals:

- Try to facilitate the team’s discussion (avoid dominating the conversation)
- Ask open-ended questions (avoid yes/no questions)
- Discuss the team performance (not the individual)

1) Introduction

This “sets the stage” for debriefing and creates expectations. What you might say:

- This is an opportunity to reflect and learn, improve our medical care, teamwork, and communication.
- Everyone should be able to ask questions and share their thoughts.
- Once you leave this session, we encourage open discussion of the concepts, but ask you to not to discuss individual performance.
- Remind the group of the ground rules (treat everyone with respect, maintain confidentiality).

2) Emotional experience discussion

There are a couple of camps regarding discussing emotions in debriefing simulation scenarios. One perspective is that until emotions are dealt with, it can be difficult for adult learners to “move on”: switching gears to process thoughts, actions and opportunities for improvement. Another perspective is that adult learners should process their emotions independently.

Our perspective is based on our experience with various simulation scenario debriefings at our institution. If a group or team member is emotionally charged (sad, mad or frustrated) regarding something that did

or did not happen in a scenario, it is usually difficult for the individual or the group to be actively engaged, receptive to feedback and able to promote learning, until the emotions are addressed.

An example: a medication error occurs. One team member may think it is all his/her fault. S/he may feel embarrassed, judged, etc. If he/she can verbalize this, other team members may offer different perspectives, which enable the team to process the error together, potentially identifying contributing systems issues. If the emotions are not addressed- team members may feel embarrassed, responsible and not engage in a discussion, failing to identify systems issues which led to the error.

In order to address this situation, consider normalizing the fact that emotions arise and offer the participants a safe and confidential setting to share. After normalizing, we recommend open-ended questions, such as:

- How did that feel?
- Can you tell me more?
- Why?

3) Medical management and technical skills

This portion of the discussion focuses on the medical aspects of the scenario. It’s usually more comfortable to begin with these “facts”. What you might say:

- Let’s begin by discussing medical management.
- What did you think was wrong with the patient?
- Can someone briefly summarize what happened in this scenario?
- How did you reach those conclusions?

4) Teamwork and communication (a.k.a. crew resource management, non-technical skills)

This portion of the discussion focuses on how the team worked together. It can be emotionally charged and difficult to discuss without feeling personal. The challenge is to try to generalize specifics into themes.

What you might say:

- Let’s talk about how you functioned as a team.
- What did your team do well?
- What could your team do differently next time?
- That is something I see often. Has anyone else experienced that? How have you seen that handled?

5) Summarizing

- This is your opportunity to ensure the key learning points are highlighted
- Try to identify approximately three take-home points
- You may ask the participants to identify take home points or call them out yourself.

**Medical management/technical skills examples:**

- This was a scenario of pediatric vitamin k deficiency brain bleed with the following goals:
- Effectively perform a primary and secondary survey.
- Evaluate and manage a neurologically decompensating patient including:
  - ABCs (airway, breathing, circulation)
  - Intravenous or intraosseous access
  - Broad differential diagnosis as to the etiology of the lethargic infant with bruising who decompensates into status epilepticus with apnea with a progressive exam notable for Cushing’s triad and pupil asymmetry.
    - Consider treating broadly initially for sepsis (with fluids and antibiotics and antivirals), for status with antiepileptic drugs, consider electrolyte and coagulopathic abnormalities, and treat for Vit K deficiency immediately upon suspicion for deficiency (if get history of inappropriate prophylaxis, treat prior to the abnormal coags return)

**Teamwork/communication examples:**

- Recognize need to call a code and call in specialists (if available at your institution) such as NICU and neurosurgery.
- Designate leadership and team member roles to ensure coordinated team functioning.
- Use brief or huddle to create a shared mental model for the working diagnosis and management plan.

Below are examples of learning objective based statements & questions you may use to debrief the team.

**Examples of debriefing for different learning objectives**

| Recognizing the sick infant | | |
| --- | --- | --- |
| Debriefer Script | Reference Material | Instructor Notes |
| I noticed you (were quick/took a while) to identify the patient was lethargic. This was (great/could lead to delays) since delays in recognition can result in worse clinical outcomes.   - What were your thought processes around what was occurring? - What helped/hindered you in deciding the A&P? | Risks/signs of neurologic disease in the infant:   - Mental status - Poor PO intake - Abnormal muscle movements | A key learning point is to be thoughtful about assessing the neurologic, respiratory and circulatory status of the infant, recognizing abnormal, and treating prioritizing via A,B,C,D,E. |

| Initial identification and management of the sick infant | | |
| --- | --- | --- |
| Debriefer Script | Reference Material | Instructor Notes |
| I noticed you (were quick/ could have been quicker) to recognize the patient was lethargic. This was (great/could lead to delays) in clinical stabilization.   - How did your team decide on the management priorities? - Did your team develop a complete differential diagnosis for the lethargic patient? - What helped/hindered you? - How might one most effectively engage the parent in this scenario to ensure an honest and complete history, including the birth history that is so important for this case? | Initial management of vitamin k deficiency brain bleed:   - Primary survey (ABCDE) - Vital signs - Assess mental status - Perform secondary survey and gather history - Identify lack of head trauma or signs of infection - Recognize history of no IM vitamin k at birth, history of oozing umbilical cord, bruising on body, and progressively worsening neurologic status concerning for brain bleed, with final coagulation studies diagnostic with prolonged PT/PTT and INR. - Stabilize airway, treat seizure, treat with Vitamin K and FFP. - Call for help - Transport patient to safety | Refer learner to the following references for more information:   - <https://pediatrics.aappublications.org/content/112/1/191> - Pediatric Advanced Life Support (PALS) algorithm (downloaded from the American Heart Association). |

**Examples for debriefing different teamwork learning objectives**

| **Roles and Responsibilities** | | |
| --- | --- | --- |
| Debriefer Script | Reference Material | Instructor Notes |
| Let’s talk about how you functioned as a team.  From my perspective it looked like you (*did/did not) have* a clear team leader and defined team roles. I think this is (*great/concerning)* because clear team roles can help a team function smoothly- improving how quickly interventions take place and reducing errors.   - How did you function as a team? - What did you think about your roles? | Team leader   - Clear direction, coordination, timely interventions - Foot of patient - Check-back communication for med dosing   Airway/Procedure MD   - Manage airway - Head of patient   Survey MD   - Primary, Secondary survey, pulses, reassess   Other roles   - Helper to call for help - Helper to get supplies/meds/airway equipment - Documenting (time keeper) - Check-back communication for tasks performed |  |

| **Brief and Huddle** | | |
| --- | --- | --- |
| Debriefer Script | Reference Material | Instructor Notes |
| I noticed that your team *(did/didn’t/took a while to)* (*brief prior to the initial patient assessment/huddle after the initial evaluation).* I thought this was (*great/could have helped you work better as a team*) in order to facilitate patient care.   - What *(helped/hindered)* your team from (*briefing/huddling*)? - How did that impact your team? - What could your team have done differently? - How can you make sure that *(does/doesn’t*) happen again? | The goal of a brief/huddle is to create a shared mental model. Assure all team members know what the working diagnosis is, management priorities and next steps in care.   - Everyone on the team is responsible for making this happen. Anyone can ask for a brief/huddle. Brief/huddle is usually led by team leader. - If one team member doesn’t know what’s up or what’s next- s/he is probably not alone. | Refer learner to the following references for more information:   - TeamSTEPPS: national implementation. Agency for Healthcare Research and Quality Web site. http://teamstepps.ahrq.gov. Accessed February 20, 2018. |

| **Directed call out** | | |
| --- | --- | --- |
| Debriefer Script | Reference Material | Instructor Notes |
| I noticed that you (*did/didn’t/intermittently*) used (*people’s names/roles/eye contact*) when (*calling out orders/asking for assistance*). I thought this was (*great/could have been more directed*) in order to facilitate communication.   - What did you notice about orders/questions that were asked? - How did this impact your team? | Directed call out. A tactical communication skill to assure that important orders/questions are specifically directed to one individual (rather than called out into the air).  Example:   - “Leah- what is the patient’s heart rate and respiratory rate?” - “Yen- Is the patient breathing on her own?” - “Team leader- she is now seizing” |  |

| **Closed loop communication/Check back** | | |
| --- | --- | --- |
| Debriefer Script | Reference Material | Instructor Notes |
| I noticed that you used closed-loop communication *(consistently/a lot/rarely)*. Closed-loop communication can be critical for catching errors and assuring that *(information/an order/a request)* is heard.   - How were the communication loops in the team? - How did that impact your team? - Has anyone seen problems with this in a patient resuscitation? - Has anyone seen closed loop communication prevent an error? - How could you do it differently next time? | Closed loop communication/check back is a strategy that requires verification of information. This enables the sender of the message to verify it has been heard and heard correctly. It enables the receiver to confirm what they heard is correct.   - Team leader: “Call for help.” - Helper: “I’m calling a code now.” - Team leader “Thank you, let me know when you reach the NICU.” |  |

**Medical** **Management** **Evaluation/Debriefing** **Form**

This checklist identifies core medical management / technical skills. It’s hard to discuss more than 3 of these during one debriefing session. We recommend focusing on 2-3 of these issues.

**Assessment of ABCDE’s □** Done Well **□** Needs Work

Specific comments:

*Discussion Points: What did you think of the assessment of the ABCDE’s? What could you do differently?*

**Avoiding premature diagnostic closure □** Done Well **□** Needs Work

Specific comments:

*Discussion Points: What other potential etiologies could cause these signs and symptoms? How did you decide*

*what the most likely cause was? What other studies and therapies would you pursue in the case?*

**Recognizing the diagnosis □** Done Well **□** Needs Work

Specific comments:

*Discuss Points: What are the specific signs of this diagnosis?*

**Initial management of the patient □** Done Well **□** Needs Work

Specific comments:

*Discuss Points: What’s the emergent management for this disease state? Ie: Management of airway, breathing and circulation, exposure, disability.*

**Teamwork** **and** **Communication** **Evaluation**

This checklist identifies core teamwork and communication skills. It’s hard to discuss more than 3 of these during

one debriefing session. We recommend focusing on 2-4 of these issues.

**Leader/Roles Identified & Maintained □** Done Well **□** Needs Work

Specific comments:

*Discussion Points: What helped/hindered having clear leadership and roles?*

**Directed Call out □** Done Well **□** Needs Work

Specific comments:

*Discussion Points: How were orders given- “Into the air” or directed at specific individuals? How did that impact*

*you? How could they be delivered more effectively?*

**Check back/Closed loop communication □** Done Well **□** Needs Work

Specific comments:

*Discussion Points: Describe closed loop communication.*

**Shared Mental Model □** Done Well **□** Needs Work

Specific comments:

*Discussion Points: How did team members share information/working diagnosis/management plan*

*(brief/huddle)?*

1. This appendix is modified from the following publications: Burns C, Burns R, Sanseau E, et al. Pediatric Emergency Medicine Simulation Curriculum: marijuana ingestion. MedEdPORTAL. 2018;14:10780. [https://doi.org/10.15766/mep_2374-8265.10780](https://urldefense.proofpoint.com/v2/url?u=https-3A__doi.org_10.15766_mep-5F2374-2D8265.10780&d=DwMFAg&c=aBkXpkKi7gN5fe5MqrMaN-VmRugaRb1IDRfSv2xVRy0&r=Zx2iyATIJOOXMNS8WfLX9ef6Yi-JO8Qtd54uueEE-x5Dt22FHK8m_DhwgS1AZ-yW&m=CwIDDYfUMT7-K3l96hsD96zzDsxriCm8MtlEZ3ewgqc&s=T9DG67R5U6nkQtOTo6dadfXf3SQAr-f1JOmmASwH9xI&e=) & Sanseau E, Reid J, Stone K, Burns R, Uspal N. Pediatric simulation cases for primary care providers: asthma, anaphylaxis, seizure in the office. MedEdPORTAL. 2018;14:10762. <https://doi.org/10.15766/mep_2374-8265.10762>. [↑](#footnote-ref-1)
