# Supplementary material for: Pediatric Emergency Medicine Simulation Curriculum: Vitamin K Deficiency in the Newborn
Source: MedEdPORTAL. 2021 Jan 25;17:11078. doi: 10.15766/mep_2374-8265.11078 (PMC7830750; doi:10.15766/mep_2374-8265.11078)
Supplement: Supplementary file 1 — VKDB Simulation Case.docxVKDB Sim Environment Preparation for Facilitator.docxVKDB Labs Imaging.docxVKDB Critical Action Checklist.docxVKDB Debrief.docxVKDB TeamSTEPPS.docxVKDB Didactic PowerPoint.pptxVKDB Handout.docxVKDB Standardized Patient Script.docxVKDB Postsim Survey.docx [file mep_2374-8265.11078-s001.zip › F. VKDB TeamSTEPPS.docx]

**Teamwork and Communication (TeamSTEPPS) Glossary^[[1]](#footnote-1)^**

A guide to communication safety process in simulation training.

This terminology can be reviewed with learners either pre-simulation or in the post-simulation debrief.

| **Term** | **Definition** |
| --- | --- |
| **Adaptability** | The ability to adjust strategies and alter a course of action in response to changing conditions (internal and external). |
| **Brief** | Discussion prior to start that assigns essential roles, establishes expectation, anticipated outcomes and likely contingencies. |
| **Call-Out** | A tactic used to communicate critical information during an emergent event. Helps the team prepare for vital next steps in patient care. *(Example: “Airway status?” – “Airway clear”; “Breath sounds?” – “Breath sounds decreased on right”)* |
| **Check-Back** | A communication strategy that requires a verification of information. The sender initiates the message; the receiver accepts it and restates the message. In return, the sender verifies that the re-statement of the original message is correct or amends if not. (*Example: “Give Benadryl 25 mg IV push” – “Benadryl 25 mg IV push” – “That’s correct”)* |
| **CUS** | Signal phrases that denote “I am **C**oncerned, I am **U**ncomfortable, This is a **S**afety Issue.” When spoken, all team members will understand clearly not only the issue but also the magnitude of the issue. |
| **Debrief** | Brief, informal information exchange session designed to improve team performance and effectiveness. |
| **DESC Script** | A technique for managing and resolving conflict. **D**escribe the specific situation or behavior; provide concrete data. **E**xpress how the situation makes you feel/what your concerns are. **S**uggest other alternatives and seek agreement. **C**onsequences should be stated in terms of impact on established team goals; strive for consensus. |
| **Huddle** | Ad hoc planning to re-establish Situational Awareness; designed to reinforce plans already in place and assess the need to adjust the plan. |
| **SBAR** | A framework for team members to structure information when communicating to one another. **S** = Situation (What is going on with the patient?) **B** = Background (What is the clinical background or context?) **A** = Assessment (What do I think the problem is?) **R** = Recommendation (What would I do to correct it?) |
| **Shared Mental Model** | An organizing knowledge structure of relevant facts and relationships about a task or situation that are commonly held by team members. |
| **Situational Awareness** | The ability to identify, process, and comprehend the critical elements of information about what is happening to the team with regards to the mission. It’s knowing “What is going around you” and “What is likely to happen next”. |
| **Situation**  **Monitoring** | The process of actively scanning and assessing elements of the situation to gain information or maintain an accurate awareness or understanding of the situation in which the team functions. |
| **Two-Challenge**  **Rule** | Assertively voicing concern at least two times to ensure it has been heard. |

1. TeamSTEPPS: national implementation. Agency for Healthcare Research and Quality Web site. http://teamstepps.ahrq.gov. Accessed February 20, 2018. [↑](#footnote-ref-1)
