# Supplementary material for: Pediatric Emergency Medicine Simulation Curriculum: Vitamin K Deficiency in the Newborn
Source: MedEdPORTAL. 2021 Jan 25;17:11078. doi: 10.15766/mep_2374-8265.11078 (PMC7830750; doi:10.15766/mep_2374-8265.11078)
Supplement: Supplementary file 1 — VKDB Simulation Case.docxVKDB Sim Environment Preparation for Facilitator.docxVKDB Labs Imaging.docxVKDB Critical Action Checklist.docxVKDB Debrief.docxVKDB TeamSTEPPS.docxVKDB Didactic PowerPoint.pptxVKDB Handout.docxVKDB Standardized Patient Script.docxVKDB Postsim Survey.docx [file mep_2374-8265.11078-s001.zip › G. VKDB Didactic PowerPoint.pptx]

## Slide 1
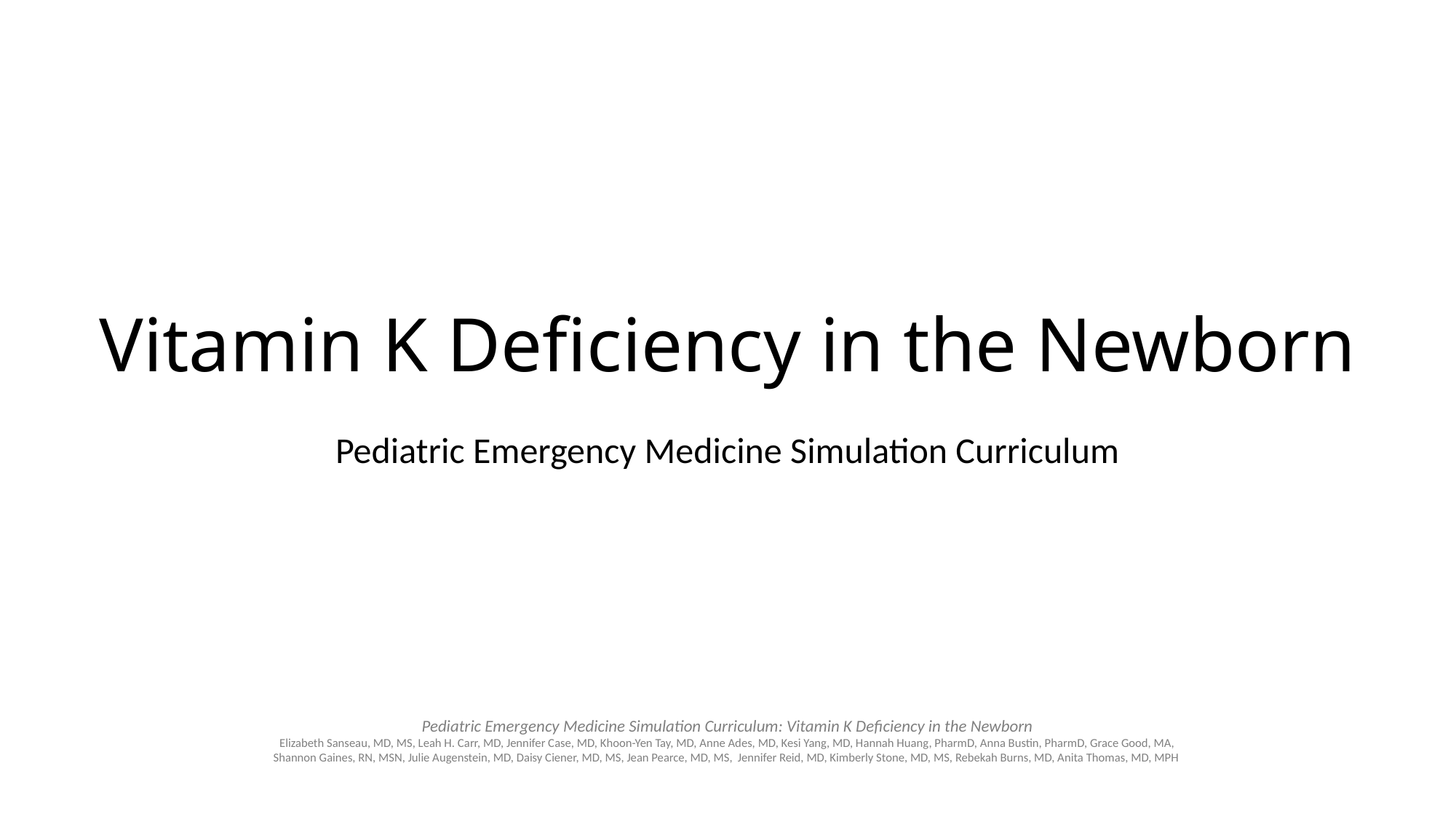

# Vitamin K Deficiency in the Newborn
Pediatric Emergency Medicine Simulation Curriculum
Pediatric Emergency Medicine Simulation Curriculum: Vitamin K Deficiency in the Newborn
Elizabeth Sanseau, MD, MS, Leah H. Carr, MD, Jennifer Case, MD, Khoon-Yen Tay, MD, Anne Ades, MD, Kesi Yang, MD, Hannah Huang, PharmD, Anna Bustin, PharmD, Grace Good, MA, Shannon Gaines, RN, MSN, Julie Augenstein, MD, Daisy Ciener, MD, MS, Jean Pearce, MD, MS, Jennifer Reid, MD, Kimberly Stone, MD, MS, Rebekah Burns, MD, Anita Thomas, MD, MPH

## Slide 2
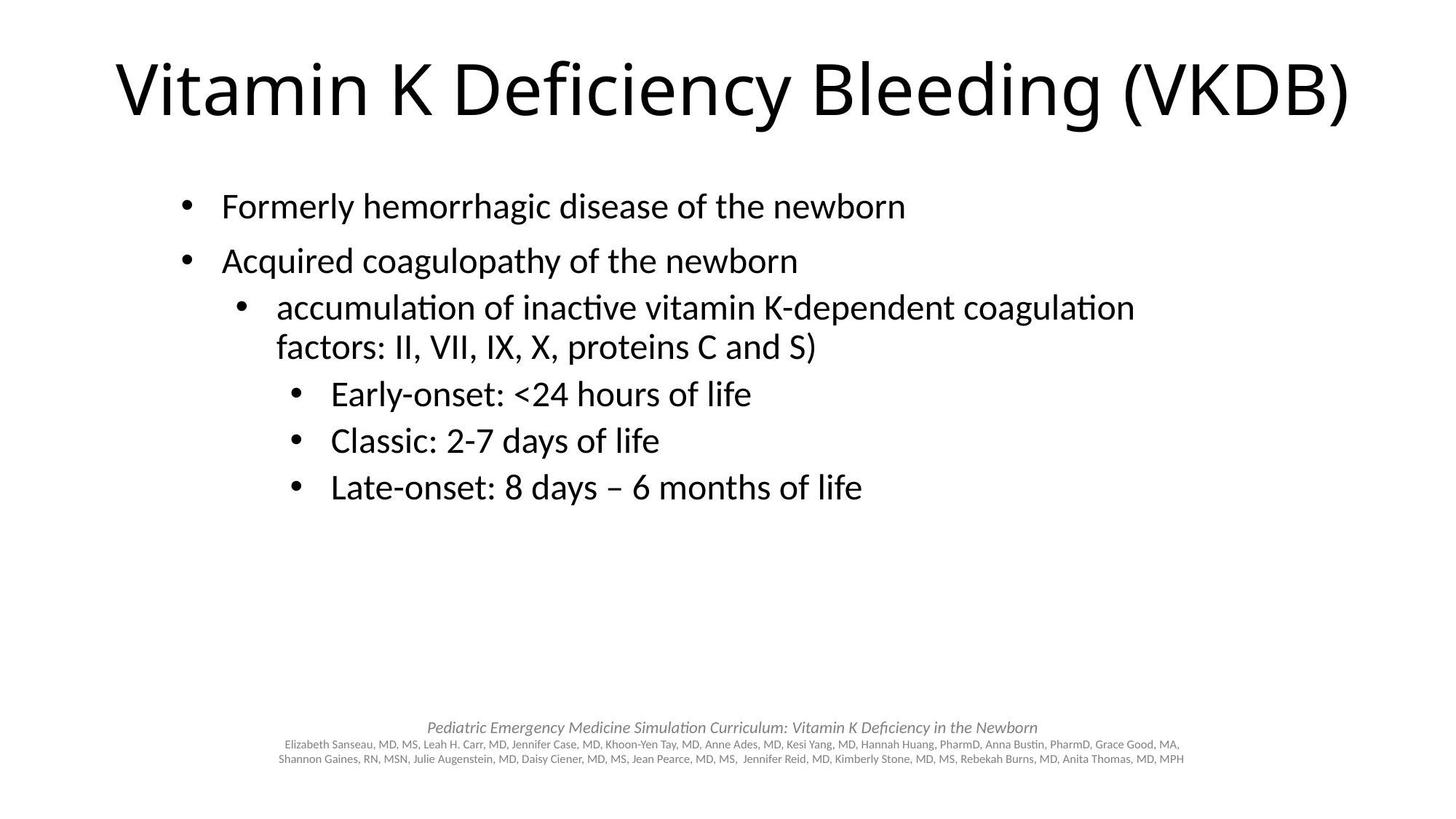

# Vitamin K Deficiency Bleeding (VKDB)
Formerly hemorrhagic disease of the newborn
Acquired coagulopathy of the newborn
accumulation of inactive vitamin K-dependent coagulation factors: II, VII, IX, X, proteins C and S)
Early-onset: <24 hours of life
Classic: 2-7 days of life
Late-onset: 8 days – 6 months of life
Pediatric Emergency Medicine Simulation Curriculum: Vitamin K Deficiency in the Newborn
Elizabeth Sanseau, MD, MS, Leah H. Carr, MD, Jennifer Case, MD, Khoon-Yen Tay, MD, Anne Ades, MD, Kesi Yang, MD, Hannah Huang, PharmD, Anna Bustin, PharmD, Grace Good, MA, Shannon Gaines, RN, MSN, Julie Augenstein, MD, Daisy Ciener, MD, MS, Jean Pearce, MD, MS, Jennifer Reid, MD, Kimberly Stone, MD, MS, Rebekah Burns, MD, Anita Thomas, MD, MPH

## Slide 3
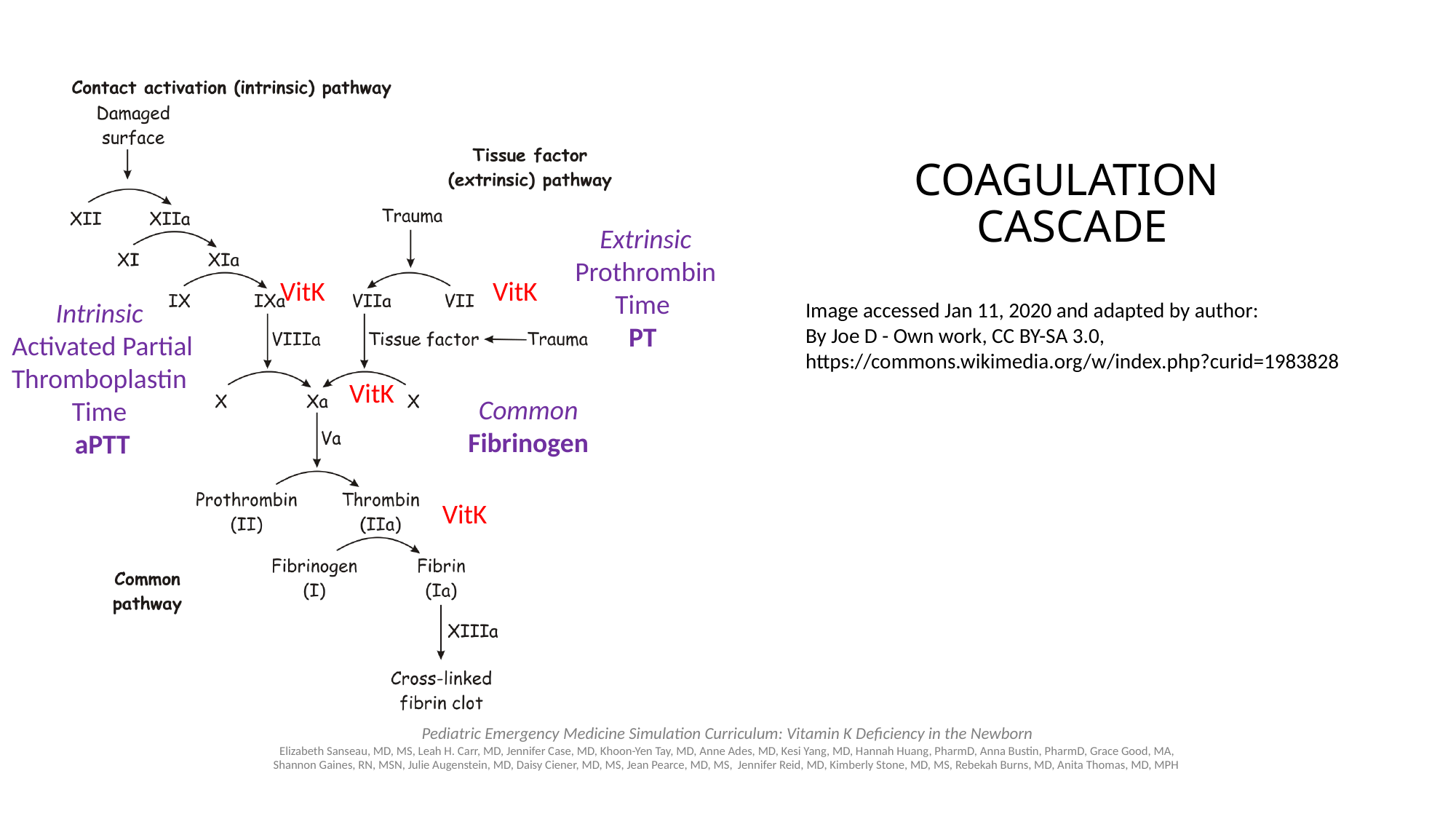

# COAGULATION CASCADE
Extrinsic
Prothrombin Time
PT
VitK
VitK
Intrinsic
Activated Partial
Thromboplastin
Time
aPTT
Image accessed Jan 11, 2020 and adapted by author:
By Joe D - Own work, CC BY-SA 3.0,
https://commons.wikimedia.org/w/index.php?curid=1983828
VitK
Common
Fibrinogen
VitK
Pediatric Emergency Medicine Simulation Curriculum: Vitamin K Deficiency in the Newborn
Elizabeth Sanseau, MD, MS, Leah H. Carr, MD, Jennifer Case, MD, Khoon-Yen Tay, MD, Anne Ades, MD, Kesi Yang, MD, Hannah Huang, PharmD, Anna Bustin, PharmD, Grace Good, MA, Shannon Gaines, RN, MSN, Julie Augenstein, MD, Daisy Ciener, MD, MS, Jean Pearce, MD, MS, Jennifer Reid, MD, Kimberly Stone, MD, MS, Rebekah Burns, MD, Anita Thomas, MD, MPH

## Slide 4
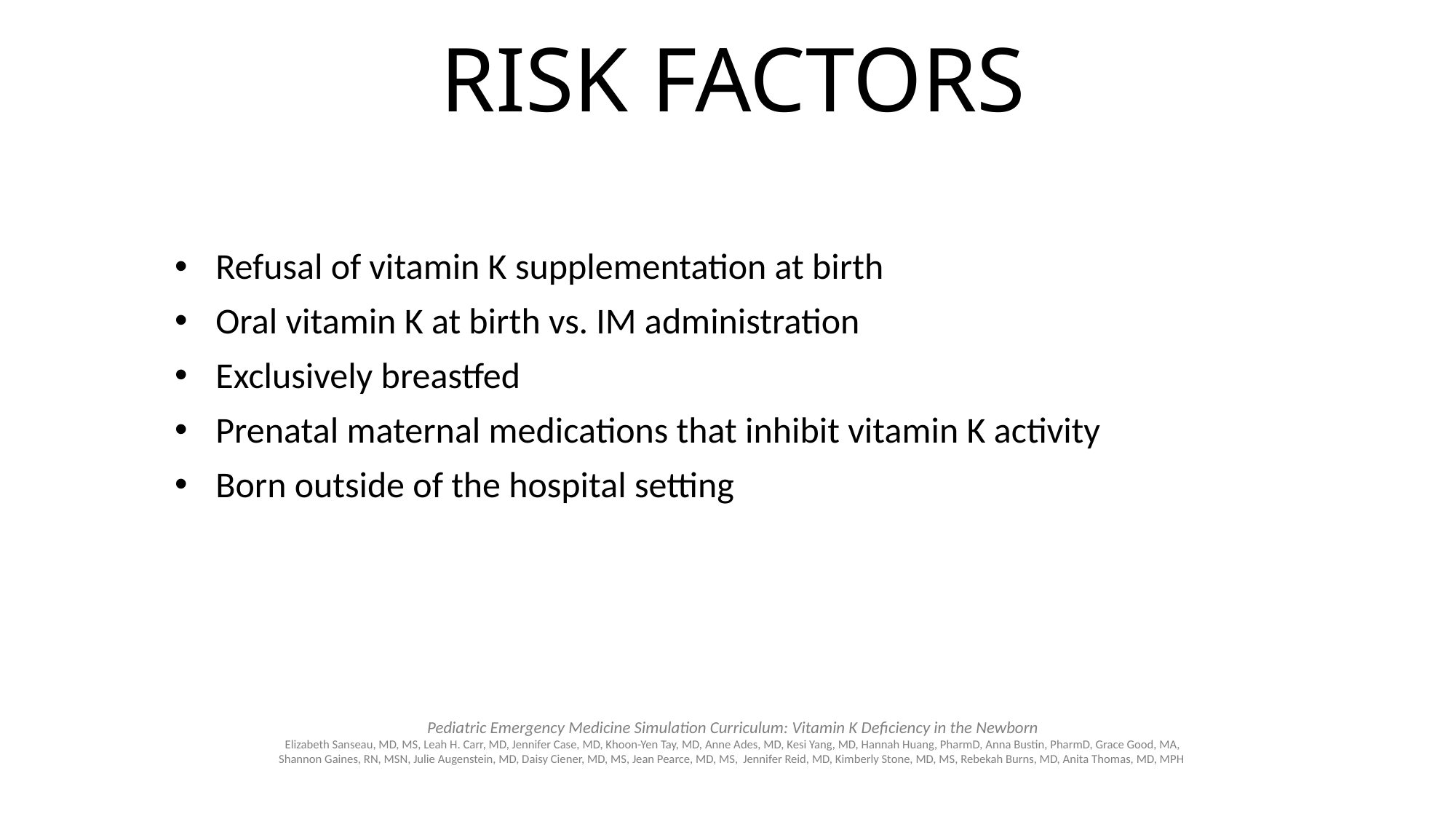

# RISK FACTORS
Refusal of vitamin K supplementation at birth
Oral vitamin K at birth vs. IM administration
Exclusively breastfed
Prenatal maternal medications that inhibit vitamin K activity
Born outside of the hospital setting
Pediatric Emergency Medicine Simulation Curriculum: Vitamin K Deficiency in the Newborn
Elizabeth Sanseau, MD, MS, Leah H. Carr, MD, Jennifer Case, MD, Khoon-Yen Tay, MD, Anne Ades, MD, Kesi Yang, MD, Hannah Huang, PharmD, Anna Bustin, PharmD, Grace Good, MA, Shannon Gaines, RN, MSN, Julie Augenstein, MD, Daisy Ciener, MD, MS, Jean Pearce, MD, MS, Jennifer Reid, MD, Kimberly Stone, MD, MS, Rebekah Burns, MD, Anita Thomas, MD, MPH

## Slide 5
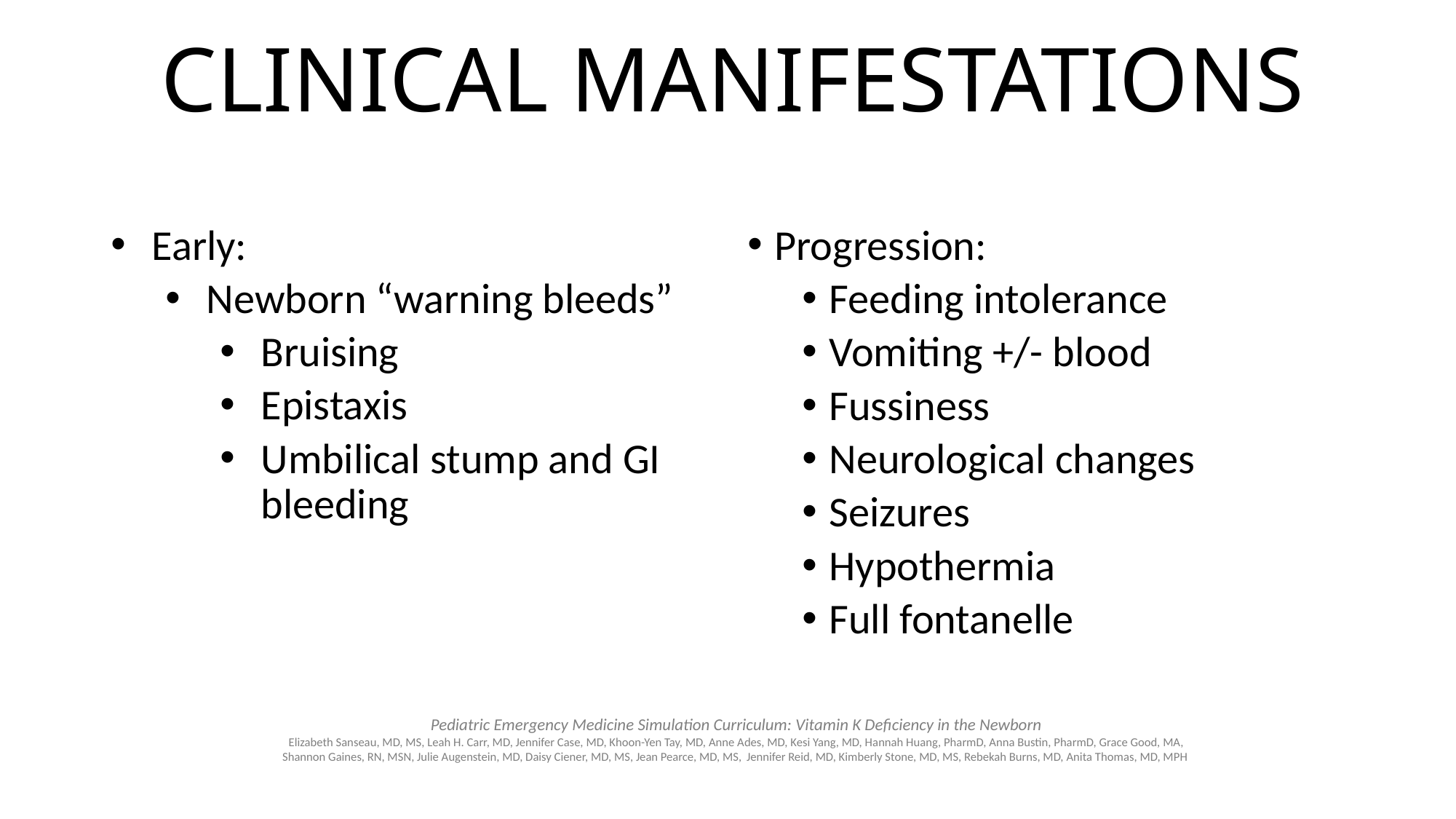

# CLINICAL MANIFESTATIONS
Early:
Newborn “warning bleeds”
Bruising
Epistaxis
Umbilical stump and GI bleeding
Progression:
Feeding intolerance
Vomiting +/- blood
Fussiness
Neurological changes
Seizures
Hypothermia
Full fontanelle
Pediatric Emergency Medicine Simulation Curriculum: Vitamin K Deficiency in the Newborn
Elizabeth Sanseau, MD, MS, Leah H. Carr, MD, Jennifer Case, MD, Khoon-Yen Tay, MD, Anne Ades, MD, Kesi Yang, MD, Hannah Huang, PharmD, Anna Bustin, PharmD, Grace Good, MA, Shannon Gaines, RN, MSN, Julie Augenstein, MD, Daisy Ciener, MD, MS, Jean Pearce, MD, MS, Jennifer Reid, MD, Kimberly Stone, MD, MS, Rebekah Burns, MD, Anita Thomas, MD, MPH

## Slide 6
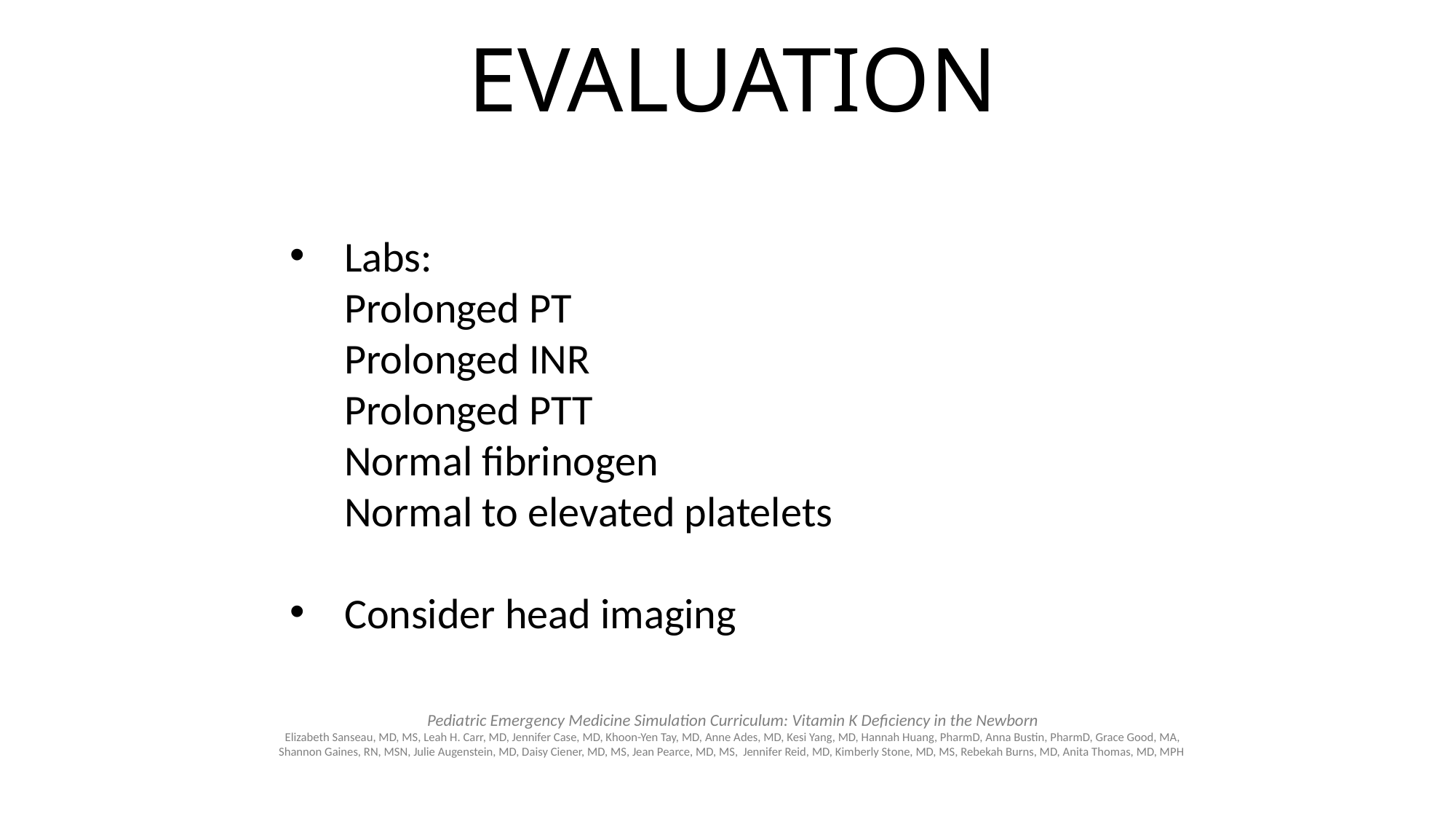

# EVALUATION
Labs:
Prolonged PT
Prolonged INR
Prolonged PTT
Normal fibrinogen
Normal to elevated platelets
Consider head imaging
Pediatric Emergency Medicine Simulation Curriculum: Vitamin K Deficiency in the Newborn
Elizabeth Sanseau, MD, MS, Leah H. Carr, MD, Jennifer Case, MD, Khoon-Yen Tay, MD, Anne Ades, MD, Kesi Yang, MD, Hannah Huang, PharmD, Anna Bustin, PharmD, Grace Good, MA, Shannon Gaines, RN, MSN, Julie Augenstein, MD, Daisy Ciener, MD, MS, Jean Pearce, MD, MS, Jennifer Reid, MD, Kimberly Stone, MD, MS, Rebekah Burns, MD, Anita Thomas, MD, MPH

## Slide 7
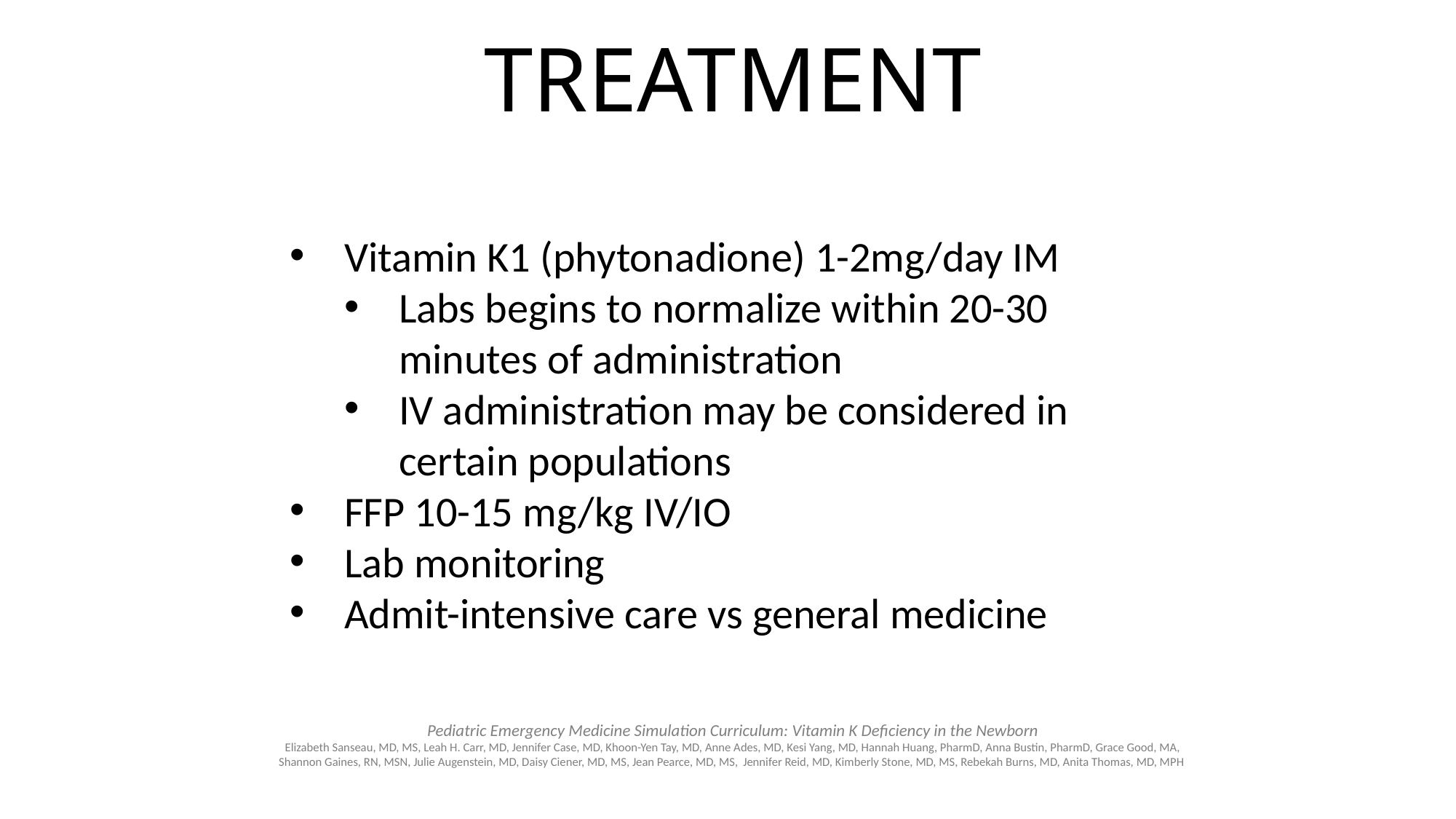

# TREATMENT
Vitamin K1 (phytonadione) 1-2mg/day IM
Labs begins to normalize within 20-30 minutes of administration
IV administration may be considered in certain populations
FFP 10-15 mg/kg IV/IO
Lab monitoring
Admit-intensive care vs general medicine
Pediatric Emergency Medicine Simulation Curriculum: Vitamin K Deficiency in the Newborn
Elizabeth Sanseau, MD, MS, Leah H. Carr, MD, Jennifer Case, MD, Khoon-Yen Tay, MD, Anne Ades, MD, Kesi Yang, MD, Hannah Huang, PharmD, Anna Bustin, PharmD, Grace Good, MA, Shannon Gaines, RN, MSN, Julie Augenstein, MD, Daisy Ciener, MD, MS, Jean Pearce, MD, MS, Jennifer Reid, MD, Kimberly Stone, MD, MS, Rebekah Burns, MD, Anita Thomas, MD, MPH
