# Supplementary material for: Pediatric Emergency Medicine Simulation Curriculum: Vitamin K Deficiency in the Newborn
Source: MedEdPORTAL. 2021 Jan 25;17:11078. doi: 10.15766/mep_2374-8265.11078 (PMC7830750; doi:10.15766/mep_2374-8265.11078)
Supplement: Supplementary file 1 — VKDB Simulation Case.docxVKDB Sim Environment Preparation for Facilitator.docxVKDB Labs Imaging.docxVKDB Critical Action Checklist.docxVKDB Debrief.docxVKDB TeamSTEPPS.docxVKDB Didactic PowerPoint.pptxVKDB Handout.docxVKDB Standardized Patient Script.docxVKDB Postsim Survey.docx [file mep_2374-8265.11078-s001.zip › H. VKDB Handout.docx]

**Definition**: *Vitamin K Deficiency Bleeding (VKDB):* previously known as Hemorrhagic Disease of the Newborn is an acquired coagulopathy due to limited Vitamin K stores at birth that presents with bleeding at 3 defined onsets:

Early: <24 hours of life

Classical: 2-7 days of life

Late: 8 days to 6 months of life

**Risk Factors:** All newborns are born with low Vitamin K stores due to poor placental transfer, therefore administration of IM vitamin K at birth is recommended for prevention however it is occasionally refused by parents causing a higher risk of bleeding

Early: maternal medication use that interferes with vitamin K storage such as antibiotics, antiepileptics and anticoagulants

Late: lack of Vitamin K injection at birth, exclusively breast fed babies

**Clinical presentation:** Symptoms can occur any time before 6 months of age and vary depending on age of onset.

Early: tend to be more severe and acute onset

Classical: can have a “warning bleed” followed by symptoms of GI or intracranial bleeding

Late: 30-60% present with intracranial bleeding and neurologic changes

**Classic symptoms:** Feeding intolerance**,** Fussiness**,** Vomiting**,** Lethargy**,** Seizures

**Warning Bleeds:** Umbilical stump or GI bleed, Bruising, Epistaxis

**Treatment:**

- Phytonadione (vitamin K) 1-2 mg/day IM (*give prior to lab results if high suspicion*)
  - Intramuscular route of administration is preferred as it is readily absorbed
  - Onset of action for IV administration ~1-2 hours, with peak effect 12-14 hours after administration
  - Intravenous administration can be considered in certain patient populations (i.e. critically ill neonates and infants)
    - Rare hypersentivity reactions have been reported with parenteral administration (IM and IV) with larger doses, faster administration time, and products that contain polyethoxylated castor oil [US boxed warning]
- FFP 10-15 mg/kg IV/IO (if patient is seizing, has brain bleed, or needs surgery)

***Crucial:***

1. Complete primary and secondary survey
2. Recognize and evaluate decompensating neurologic exam into status and consider intracranial etiology.
3. Involve NICU/neurosurgery team
4. Obtain IO access after failing IV attempts
5. Secure patient’s airway
6. Manage seizure
7. Recognize and address patient’s risk of bleeding diathesis with vitamin K and FFP
8. Consider broad differential diagnosis including: coagulopathy, inflicted or accidental trauma, sepsis, inborn error of metabolism, seizure disorder, intracranial lesion, etc.

***Second line:***

1. Assign clear team roles and responsibilities
2. Communicate effectively as a team, including using directed, closed-loop communication
3. Comfort patient’s parent
4. Give clear recap of patient’s history to consulting team
