# Supplementary material for: Pediatric Emergency Medicine Simulation Curriculum: Vitamin K Deficiency in the Newborn
Source: MedEdPORTAL. 2021 Jan 25;17:11078. doi: 10.15766/mep_2374-8265.11078 (PMC7830750; doi:10.15766/mep_2374-8265.11078)
Supplement: Supplementary file 1 — VKDB Simulation Case.docxVKDB Sim Environment Preparation for Facilitator.docxVKDB Labs Imaging.docxVKDB Critical Action Checklist.docxVKDB Debrief.docxVKDB TeamSTEPPS.docxVKDB Didactic PowerPoint.pptxVKDB Handout.docxVKDB Standardized Patient Script.docxVKDB Postsim Survey.docx [file mep_2374-8265.11078-s001.zip › J. VKDB Postsim Survey.docx]

**Neonatal Vitamin K Deficiency Simulation Session Evaluation Form**

**Instructor:** ____________________________ **Date:**  _________________

**Circle One**: Medical student, Resident (peds, med-peds, EM, FM)/Fellow (PEM, NICU, Critical Care, Neurosurgical, other)/Attending

**Case Presented:** Neonatal Vitamin K Deficiency

|  | Strongly  Disagree | Disagree | Neutral | Agree | Strongly  Agree |
| --- | --- | --- | --- | --- | --- |
| 1. This simulation case provided is relevant to my work. | 1 | 2 | 3 | 4 | 5 |
| 1. The simulation case was realistic. | 1 | 2 | 3 | 4 | 5 |
| 1. This simulation case was effective in teaching recognition of neonatal vitamin K deficiency. | 1 | 2 | 3 | 4 | 5 |
| 1. I feel prepared to stabilize a patient with neonatal vitamin K deficiency. | 1 | 2 | 3 | 4 | 5 |
| 1. This simulation case was effective in teaching management of neonatal vitamin K deficiency. | 1 | 2 | 3 | 4 | 5 |
| 1. I feel comfortable activating team assistance early in a resuscitative event. | 1 | 2 | 3 | 4 | 5 |
| 1. This scenario allowed me to practice effective teamwork and communication skills. | 1 | 2 | 3 | 4 | 5 |
| 1. The debrief created a safe environment. | 1 | 2 | 3 | 4 | 5 |
| 1. The debrief promoted reflection and team discussion. | 1 | 2 | 3 | 4 | 5 |

Can you list/describe 1 or more ways this simulation session will change how you do your job

How can we improve this scenario?

Comments:
