# Supplementary material for: Pediatric Emergency Medicine Simulation Curriculum: Vitamin K Deficiency in the Newborn
Source: MedEdPORTAL. 2021 Jan 25;17:11078. doi: 10.15766/mep_2374-8265.11078 (PMC7830750; doi:10.15766/mep_2374-8265.11078)
Supplement: Supplementary file 1 — VKDB Simulation Case.docxVKDB Sim Environment Preparation for Facilitator.docxVKDB Labs Imaging.docxVKDB Critical Action Checklist.docxVKDB Debrief.docxVKDB TeamSTEPPS.docxVKDB Didactic PowerPoint.pptxVKDB Handout.docxVKDB Standardized Patient Script.docxVKDB Postsim Survey.docx [file mep_2374-8265.11078-s001.zip › I. VKDB Standardized Patient Script.docx]

STANDARDIZED PATIENT (SP) SCRIPT

*This script is to be used by a SP playing the mother who just brought in her 4wk old baby into your Emergency Room (ER) due to lethargy and bruising.*

CASE BACKGROUND INFORMATION

*The case is a 4-week-old, ex-full term, baby girl brought in by her mother to the pediatric ER due to increasing fussiness, lethargy, intermittent episodes of tremulousness and bruising that has been getting worse over the past 5 days. The baby delivered at home without notable complications. Parents refused vitamin K, erythromycin and Hepatitis B at birth. She has been seen by her Naturopath pediatrician at a 2 week visit, at which point she had regained her birthweight. She is exclusively breastfeeding. Her newborn screen returned normal. Upon arrival to the ER, babe is irritable and inconsolable. As soon as the patient is placed on the exam table in the resuscitation bay, she becomes lethargic, quiet, and no longer opens her eyes. At this point, a full code is called and the mother is attended to by a physician assigned to talk with her. The patient starts seizing and requires many medical interventions, including IO access, intubation, several medications (to treat for seizure, possible sepsis, and finally for the vitamin K deficiency bleeding disorder upon discovery by participants).*

WHO ARE THE LEARNERS?

*The targeted learners are medical students, residents, fellow and faculty who would be in the position to respond to a sick infant. The level of the experience of the learner is varied among this group. Some have extensive experience coding a sick infant (i.e.: Attending physicians). Others might have less experience (i.e.: Fellow physicians). All learners do have significant experience in gathering information from patients and family members.*

STANDARDIZED PATIENT INFORMATION

*The mother is emotional about not being able to hold her baby and is frightened by the austere and unfamiliar ER environment. She should make it known that she is very upset and wants to be with her baby, but is calmed by the provider who is attending to her as to not distract the participants from the neonatal resuscitation.*

*Meanwhile, the infant is being attended to by the medical team. The baby is lethargic, tremulous, and needs a full medical resuscitation away from the mother. The medical team resuscitates the baby by doing the following: breathing for the baby, getting IO access after failing IV, treating the baby for a seizure and discovering and treating for vitamin K deficiency causing a brain bleed and eliciting prompt specialty help from the Neonatal Intensive Care Unit (NICU) and neurosurgical specialty services (if available). Labs are ordered and are notable for signs of vitamin K deficiency. Brain imaging of the baby is ordered if available (due to the abnormal neurologic status) and participants note the head bleed. The baby is treated with vitamin K, fresh frozen plasma (FFP) and transferred to the NICU prompt neurosurgical consultation.*

PATIENT INFORMATION (Please remember not to offer any of this information, but when asked please respond while remaining in character.)

CHIEF COMPLAINT: *Lethargic with scattered bruising.*

NAME: *Hallie*

AGE: *4 weeks old*

ADDITIONAL HISTORY: *Born at home, did not receive erythromycin eye drops, hepatitis B vaccine or vitamin K prophylaxis.*

PAST MEDICAL HISTORY: *None. At one week checkup did notice some oozing at umbilical stump, but primary care physician said there was nothing to worry about.*

SOCIAL HISTORY: *Lives with Mom, Dad at home. Grandparents watch baby at times, nobody else. No concerns for inflicted or accidental trauma. Followed by a Naturopath.*

FAMILY HISTORY: *No known history of coagulation or bleeding/hematologic disorders.*

PAST SURGICAL HISTORY: *None.*

MEDICATIONS: *None.*

ALLERGIES: *None.*

IMMUNIZATIONS: *None.*

PRENATAL HISTORY: *Normal, no maternal meds.*

POTENTIAL DIALOGUE:

REMEMBER, IT IS IMPORTANT THAT YOU DO NOT OFFER INFORMATION. PLEASE ALLOW THE LEARNERS TO ASK QUESTIONS. **DO NOT OFFER INFORMATION UNLESS THEY ASK YOU**.

THINGS YOU COULD SAY WITHOUT BEING ASKED:

*How is my baby? I want to hold my baby. Is my baby seizing? Is that bruising? Is my baby bleeding? Can someone tell me what’s going on?*

THINGS YOU MIGHT SAY TRIGGERED BY EVENTS IN THE SCENARIO.

*Are those medications you’re giving my baby necessary?*

*If asked about who is with the baby and if you’re concerned about trauma, say only you and your husband and the grandparents have been with her and you have no concerns.*

*When prompted, reiterate that no shots were given at the home birth and that you don’t like toxic medications.*
